# ARID1A-deficient bladder cancer is dependent on PI3K signaling and sensitive to EZH2 and PI3K inhibitors

**DOI:** 10.1172/jci.insight.155899

**Published:** 2022-08-22

**Authors:** Hasibur Rehman, Darshan S. Chandrashekar, Chakravarthi Balabhadrapatruni, Saroj Nepal, Sai Akshaya Hodigere Balasubramanya, Abigail K. Shelton, Kasey R. Skinner, Ai-Hong Ma, Ting Rao, Sumit Agarwal, Marie-Lisa Eich, Alyncia D. Robinson, Gurudatta Naik, Upender Manne, George J. Netto, C. Ryan Miller, Chong-xian Pan, Guru Sonpavde, Sooryanarayana Varambally, James E. Ferguson

**Affiliations:** 1 Department of Urology,; 2 O’Neal Comprehensive Cancer Center,; 3 Department of Pathology, and; 4 Informatics Institute, University of Alabama at Birmingham, Birmingham, Alabama, USA.; 5 Neuroscience Curriculum, University of North Carolina at Chapel Hill, Chapel Hill, North Carolina, USA.; 6 Department of Biochemistry and Molecular Medicine, School of Medicine, University of California, Davis, Sacramento, California, USA.; 7 Department of Urology, Renmin Hospital of Wuhan University, Wuhan, China.; 8 Institute of Pathology, University Hospital Cologne, Cologne, Germany.; 9 Department of Medicine, Lank Center for Genitourinary Oncology, and; 10 Department of Medicine, Dana-Farber Cancer Institute, Harvard Medical School Boston, Massachusetts, USA.; 11 Birmingham Veterans Affairs Medical Center, Birmingham, Alabama, USA.

**Keywords:** Oncology, Urology

## Abstract

Metastatic urothelial carcinoma is generally incurable with current systemic therapies. Chromatin modifiers are frequently mutated in bladder cancer, with *ARID1A*-inactivating mutations present in about 20% of tumors. EZH2, a histone methyltransferase, acts as an oncogene that functionally opposes ARID1A. In addition, PI3K signaling is activated in more than 20% of bladder cancers. Using a combination of in vitro and in vivo data, including patient-derived xenografts, we show that ARID1A-mutant tumors were more sensitive to EZH2 inhibition than ARID1A WT tumors. Mechanistic studies revealed that (a) ARID1A deficiency results in a dependency on PI3K/AKT/mTOR signaling via upregulation of a noncanonical PI3K regulatory subunit, PIK3R3, and downregulation of MAPK signaling and (b) EZH2 inhibitor sensitivity is due to upregulation of PIK3IP1, a protein inhibitor of PI3K signaling. We show that PIK3IP1 inhibited PI3K signaling by inducing proteasomal degradation of PIK3R3. Furthermore, ARID1A-deficient bladder cancer was sensitive to combination therapies with EZH2 and PI3K inhibitors in a synergistic manner. Thus, our studies suggest that bladder cancers with *ARID1A* mutations can be treated with inhibitors of EZH2 and/or PI3K and revealed mechanistic insights into the role of noncanonical PI3K constituents in bladder cancer biology.

## Graphical abstract

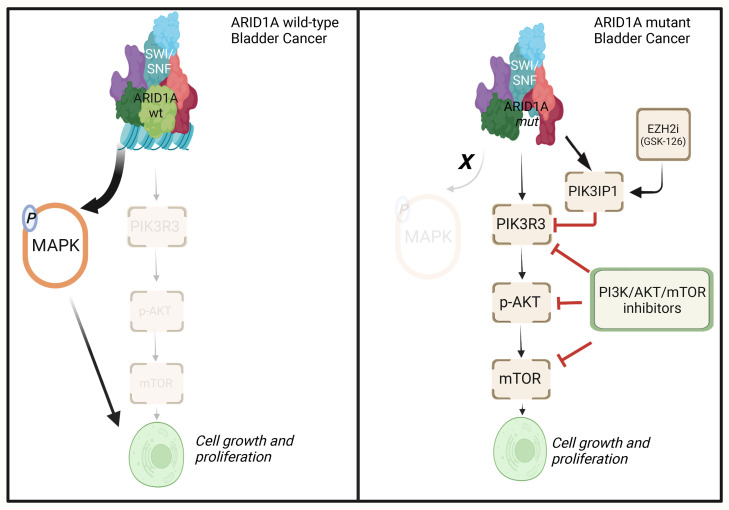

## Introduction

Bladder cancer is the sixth most common cancer in the US, and it leads to approximately 18,000 deaths annually (1). Bladder cancer outcomes have been relatively stagnant despite the recent introduction of a number of new therapies, including immune checkpoint blockade, antibody drug conjugates, and a targeted agent. Next-generation sequencing has revolutionized our understanding of bladder cancer and provides an opportunity to develop personalized therapy (2, 3). Nevertheless, so far only one targeted agent, erdafitinib, has been approved by the US FDA. Erdafitinib targets the fibroblast growth factor receptor 2/3–activating (FGFR2/3-activating) mutations or fusions that occur in less than 20% of advanced bladder cancer (4).

Sequencing data sets have revealed that genes encoding epigenetic/chromatin modifiers are frequently mutated in bladder cancer, as up to 90% of tumors have inactivating mutations in at least one chromatin-modifying enzyme (5). About 20% of bladder cancers have truncating and inactivating mutations in the AT-rich interactive domain 1A (*ARID1A*) gene, a member of the SWI/SNF chromatin modifying complex, making it one of the most frequently mutated epigenetic genes in bladder cancer. ARID1A is the DNA-binding component of the large multicomponent, 1.15 MDa SWI/SNF complex, which is important for ATP-dependent chromatin remodeling that generally results in increased transcriptional accessibility and modulates diverse gene programs and cellular processes, including DNA repair, telomere cohesion, and immune recognition (reviewed in refs. 6, 7). In human cancers, *ARID1A* shows predominantly nonsense truncating point mutations, resulting in lower protein levels and overall inactivation (6, 8). Because ARID1A is the central DNA-binding component of SWI/SNF, it is thought that these heterozygous truncating mutations deactivate the complex either through incomplete complex assembly or a dominant negative effect. Consistent with a gene dose effect from heterozygous mutations, mice with heterozygous deletion of *ARID1A* are embryonic lethal (9).

We and others have shown that the histone methyltransferase enhancer of zeste homolog 2 (EZH2) is overexpressed in many aggressive cancers, for which it is thought to drive growth and is thus considered an oncogene (10–13). EZH2 functions as the catalytic subunit of the polycomb repressive complex 2 (PRC2), which trimethylates lysine 27 on histone 3 (H3K27me3), resulting in transcriptional silencing of numerous genes, including tumor suppressors (14–16). In aggressive bladder cancers, EZH2 expression is high and promotes proliferation of bladder cancer cells (17, 18). In 2020, the EZH2 inhibitor tazemetostat received FDA approval for treatment of soft-tissue sarcomas and lymphomas (19–21).

It has been shown in various models that mutations in *ARID1A* sensitize cells to EZH2 pharmacologic inhibition with the small-molecule GSK-126 (22–24). We hypothesized that bladder cancer cells with *ARID1A* mutations would show sensitivity to EZH2 inhibition that could be utilized as a therapeutic target for patients with ARID1A-deficient bladder cancer.

Several studies suggest the cross-talk between the ARID1A and PI3K pathways in clear cell ovarian cancer and have shown that synthetic lethality by targeting EZH2 in *ARID1A*-mutated tumors correlates with inhibition of PI3K/AKT signaling (22, 25). Of the 4 classes of PI3K, class I PI3K is the main subtype that phosphorylates phospho-inositide 4,5-bisphosphate to phospho-inositide 3,4,5-triphosphate (PIP3) in various cellular membranes, activates the downstream AKT/mTOR pathway, and plays important roles in cell survival, growth, proliferation, autophagy, differentiation, and metabolism (26–30). It is the downstream signal transducer of many cell surface receptors, and abnormal activation of this pathway is often associated with oncogenesis. In bladder cancer, somatic alterations that lead to the activation of the PI3K/AKT/mTOR pathway occur in over one-third of cases (31). We have previously shown that activating alterations along the PI3K pathway are potential drivers and can possibly be targeted for the treatment of bladder cancer (32, 33).

Class I PI3K enzymes are heterodimers consisting of a catalytic subunit and a regulatory subunit (reviewed in refs. 34–36). Binding of regulatory subunits to catalytic subunits stabilizes catalytic subunit proteins and allows for precise modulation of their enzymatic activity. There are 2 widely expressed catalytic subunit proteins coded for by 2 genes, *PIK3CA*/p110α and *PIK3CB*/p110β, and 5 regulatory subunit proteins expressed from 3 genes, *PIK3R1*/p85α/p55α/p50α, *PIK3R2*/p85β, and *PIK3R3*/p55γ. Of note, *PIK3R1* produces 3 separate proteins, with identical C-termini, through alternative splicing. Although the functional ramifications of the various heterodimer configurations and protein products of the regulatory subunits *PIK3R1* and *PIK3R2* have been fairly well characterized (27, 29), comparatively little is understood about the relative contributions of *PIK3R3*/p55γ or how it interacts preferentially with the catalytic subunits p110α or p110β or potentially competes with other regulatory subunits (28). Furthermore, PI3K inhibitors with relative specificity for p110α have been developed to maximize the therapeutic ratio relative to pan–class I inhibitors. But whether they are effective against heterodimers containing *PIK3R3*/p55γ is not well understood. Our studies add significantly to the understanding of the role of PIK3R3 in PI3K biology.

Overall, our investigations reveal a molecular dependence of ARID1A-deficient bladder cancers on PI3K/AKT signaling due to upregulation of *PIK3R3* and show a synergistic antitumor activity of EZH2 and PI3K inhibitors, suggesting that this combination could be repurposed for the treatment of bladder cancer with alterations along these pathways that occur in over 40% of patients with bladder cancer.

## Results

### ARID1A-inactivating mutations in bladder cancers.

**Figure 1:**
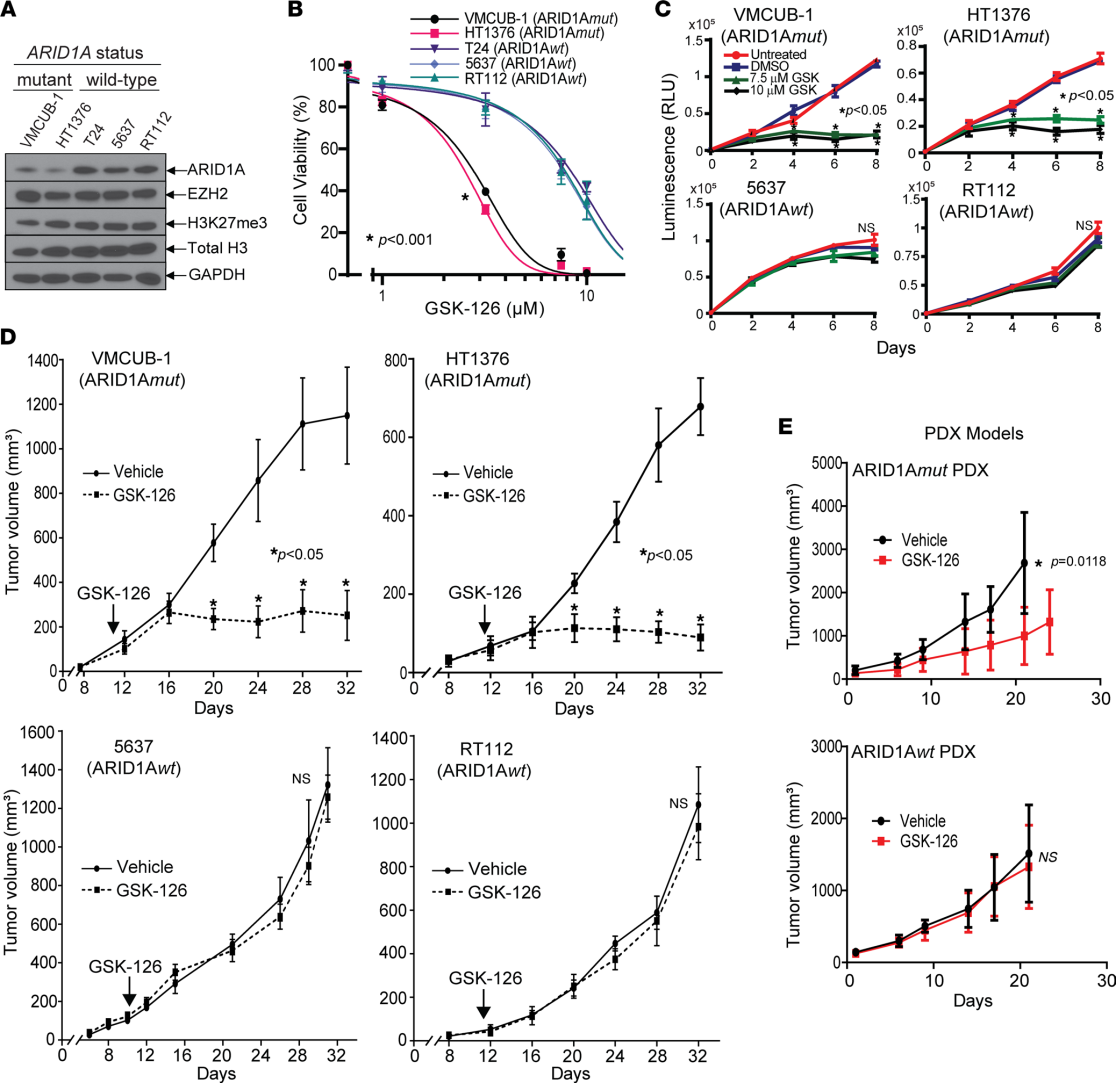
Bladder cancer cells and xenografts with inactivating ARID1A mutations are sensitive to EZH2 inhibition. (**A**) Immunoblots showing lower protein levels of ARID1A in bladder cancer cell lines harboring heterozygous *ARID1A*-truncating mutations (HT1376 and VMCUB-1), compared with ARID1A*wt* alleles (T24, 5637, and RT112). (**B**) Cell viability dose-response assay showing that ARID1A*mut* bladder cancer cells are more sensitive to the EZH2 inhibitor, GSK-126 than ARID1A*wt* cells (treated for 6 days). Two-way ANOVA using IC_50_ values was performed. (**C**) Cell viability time course with increasing concentrations of GSK-126, indicating that ARID1A*mut* cell lines are more sensitive than ARID1A*wt* cell lines. (**D**) Xenografts from ARID1A*mut* cells are sensitive to GSK-126, whereas ARID1A*wt* xenografts are resistant. (**E**) Xenografts (PDX) derived from bladder cancers with *ARID1A* mutations are more sensitive to GSK-126 than ARID1A*wt* xenografts. Unless otherwise specified, *t* tests were performed. N.S. denotes “nonspecific.”

To assess the prevalence of *ARID1A* mutations in bladder cancer, we performed in silico mutation analysis via cBioPortal (http://www.cbioportal.org/), using the Memorial Sloan Kettering Cancer Center bladder cancer sequencing data set and The Cancer Genome Atlas (TCGA) data set (2). This analysis indicated that up to 29% of bladder cancers have nonsense or truncating mutations in *ARID1A* (Supplemental Figure 1A; supplemental material available online with this article; https://doi.org/10.1172/jci.insight.155899DS1). Furthermore, *ARID1A*-mutated (herein referred to as ARID1A*mut*) bladder cancers expressed high levels of EZH2, consistent with previous findings that EZH2 expression is upregulated in bladder cancer (17) (Supplemental Figure 1B). Our analysis of the COSMIC cell line data set (https://cancer.sanger.ac.uk/cosmic) (Wellcome Trust Sanger Institute) suggested that various bladder cancer cell lines harbored mutations in *ARID1A*. Among these, HT1376 had a frameshift deletion, and VM-CUB1 had a nonsense substitution mutation. Other bladder cancer cells (T24, RT-112, and 5637) did not have mutations in the *ARID1A* gene. Furthermore, cell lines harboring truncating mutations in *ARID1A* showed lower levels of the ARID1A protein, without differences in EZH2 or EZH2 methyltransferase activity (Figure 1A).

### ARID1A mutation is associated with sensitivity to EZH2 inhibitors in vitro and in vivo.

GSK-126 is a specific small-molecule inhibitor of EZH2. To investigate its effect on proliferation of ARID1A*mut* bladder cancer cells, we performed viability and proliferation assays using cell lines with and without mutations in *ARID1A*. The IC_50_s for ARID1A*mut* bladder cancer cell lines HT1376 (2.5 μM) and VM-CUB1 (2.8 μM) were much lower than ARID1A WT (ARID1A*wt*) cell lines T24 (8.3 μM), 5637 (7.6 μM), and RT112 (7.8 μM) (Figure 1B). There were similar differences using proliferation assays (Figure 1C and Supplemental Figure 2, A and B) and colony-formation assays (Supplemental Figure 2C). To confirm that the concentrations used were effective in inhibiting the histone methyltransferase activity of EZH2, we performed immunoblot analysis for the EZH2 methyltransferase product, H3K27me3. As expected, GSK-126 lowered H3K27me3 levels in all bladder cancer cell lines (Supplemental Figure 2A). Thus, at these concentrations, GSK-126 could efficiently inhibit EZH2-dependent histone methylation, but only ARID1A*mut* bladder cancer cells were sensitive while ARID1A*wt* cells were resistant.

To determine the antitumor activity in vivo, we performed murine xenograft experiments with human ARID1A*mut* and ARID1A*wt* bladder cancer cells. Consistent with the in vitro findings, systemic GSK-126 treatment effectively inhibited histone methylation in both ARID1A*wt* and ARID1A*mut* tumors (Supplemental Figure 3A), but it only suppressed growth of ARID1A*mut* tumors but not ARID1A*wt* tumors (Figure 1D). We previously reported that patient-derived xenografts (PDXs) retain the morphology and genomic fidelities and are considered one of the best models in reflecting patient cancers’ pharmacologic susceptibilities (32). To evaluate the translational significance of this association, we tested the GSK-126 sensitivity of PDXs harboring ARID1A*wt* and mutant alleles. ARID1A*mut* PDX models were sensitive to GSK-126, but ARID1A*wt* models were resistant (Figure 1E).

### ARID1A knockdown induces GSK-126 sensitivity in ARID1Awt bladder cancer cells.

**Figure 2:**
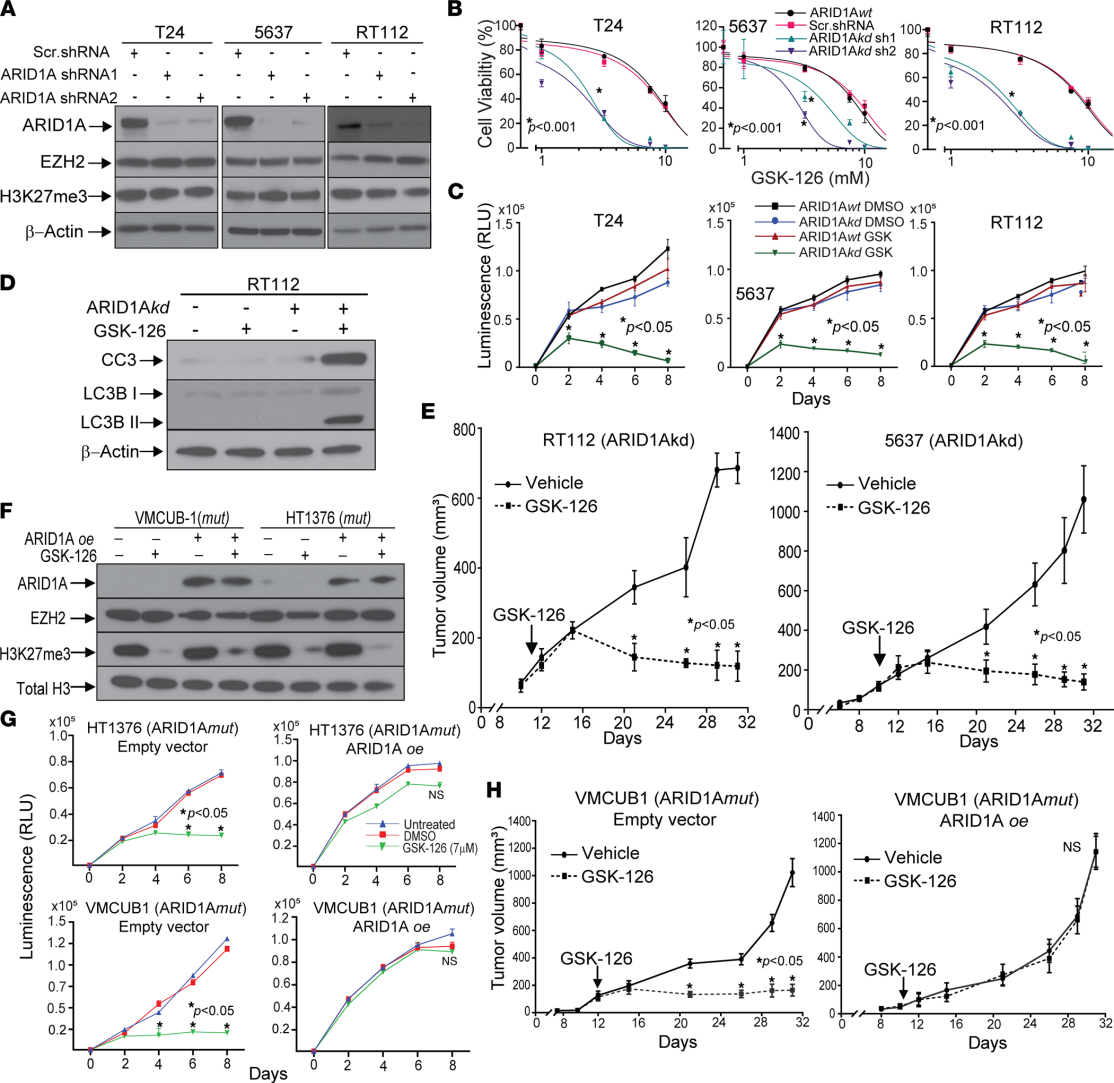
ARID1A deficiency in bladder cancer cells is necessary and sufficient for sensitivity to EZH2 inhibition. (**A**) Immunoblots showing expression of ARID1A, EZH2, and tri-methylated H3K27 (H3K27me3) in ARID1A*wt* bladder cancer cell lines after ARID1A stable knockdown (KD) with 2 separate shRNA sequences along with scrambled (scr) shRNA. (**B**) Cell viability dose-response assay showing that ARID1A*kd* bladder cancer cells are more sensitive to the EZH2 inhibitor GSK126 than scr shRNA controls (treatment for 6 days). Two-way ANOVA using IC_50_ values was performed. (**C**) Cell viability time course with GSK-126 treatment showing that ARID1A*kd* cells are more sensitive than ARID1A*wt* cells. (**D**) Immunoblot for cleaved caspase-3 (CC3) and LC3BII, indicating that apoptosis and autophagy are activated in ARID1A*kd* cells treated with GSK-126 for 48 hours. (**E**) Xenografts from mice inoculated with ARID1A*kd* cells showing that these tumors are sensitive to GSK-126 treatment. (**F**) Immunoblot analysis of ARID1A*mut* cell lines stably transduced with ARID1A overexpression (ARID1A*oe*) lentivirus or empty vector control and treated with GSK-126 for 48 hours. (**G**) Cell proliferation assays using ARID1A*mut* cell lines with or without ARID1A*oe*, indicating that sensitivity to GSK-126 is abrogated by ARID1A reconstitution. (**H**) Xenografts from ARID1A*mut* cell lines with ARID1A*oe* are resistant to GSK-126 inhibition. Unless otherwise specified, *t* tests were performed. N.S. denotes “non-specific.”

To mimic the functional ramifications of inactivating mutations in a more genetically defined model, we generated shRNA-mediated stable knockdown of ARID1A in all 3 ARID1A*wt* cell lines (herein referred to as ARID1A*kd* cells) (Figure 2A). Although ARID1A knockdown did not affect cell viability/proliferation at baseline, it did result in higher sensitivity to GSK-126, as evidenced by lower viability (Figure 2B), decreased proliferation (Figure 2C), and decreased colony formation (Supplemental Figure 4A). Furthermore, after treatment with GSK-126, apoptosis and autophagy were activated in ARID1A*kd* cells, but not in ARID1A*wt* cells, as evidenced by increased cleaved caspase-3 and LC3BII immunoblotting (Figure 2D). These ARID1A*kd* cells were then used to generate xenografts to test their sensitivity to EZH2 inhibition in vivo. Although RT112 and 5637 ARID1A*wt* xenografts were resistant to GSK-126 (Figure 1D), ARID1A*kd* cells were sensitive, and their growth was nearly completely inhibited (Figure 2E and Supplemental Figure 4B). To confirm that EZH2 inhibitor sensitivity was generalizable to other drugs, we performed these dose-response cell viability experiments using 3 different EZH2 inhibitors: CPI-1205 and EPZ-6438, both SAM-specific inhibitors that inhibit EZH2 catalytic activity in a manner similar to GSK-126, and MAK683, an EED binding inhibitor that disrupts the PRC2 complex, leading to EZH2 protein degradation. Similar results were found (Supplemental Figure 4, C and D).

Next, we performed reconstitution experiments wherein ARID1A*mut* cells were stably transduced with ARID1A-expressing constructs via lentivirus (Figure 2F). Cell viability assays showed that ARID1A overexpression/reconstitution induced GSK-126 resistance (Figure 2G). Activity of GSK-126 was confirmed by immunoblot for H3K27Me3 (Figure 2F). Xenograft studies showed that ARID1A-reconstituted mutant cell lines were resistant to GSK-126 in vivo (Figure 2H and Supplemental Figure 4E). Thus, we confirmed that sensitivity of bladder cancer cells to EZH2 inhibition is dependent on ARID1A deficiency.

### Investigating the mechanisms of EZH2 sensitivity in ARID1A-deficient bladder cancer cells.

**Figure 3:**
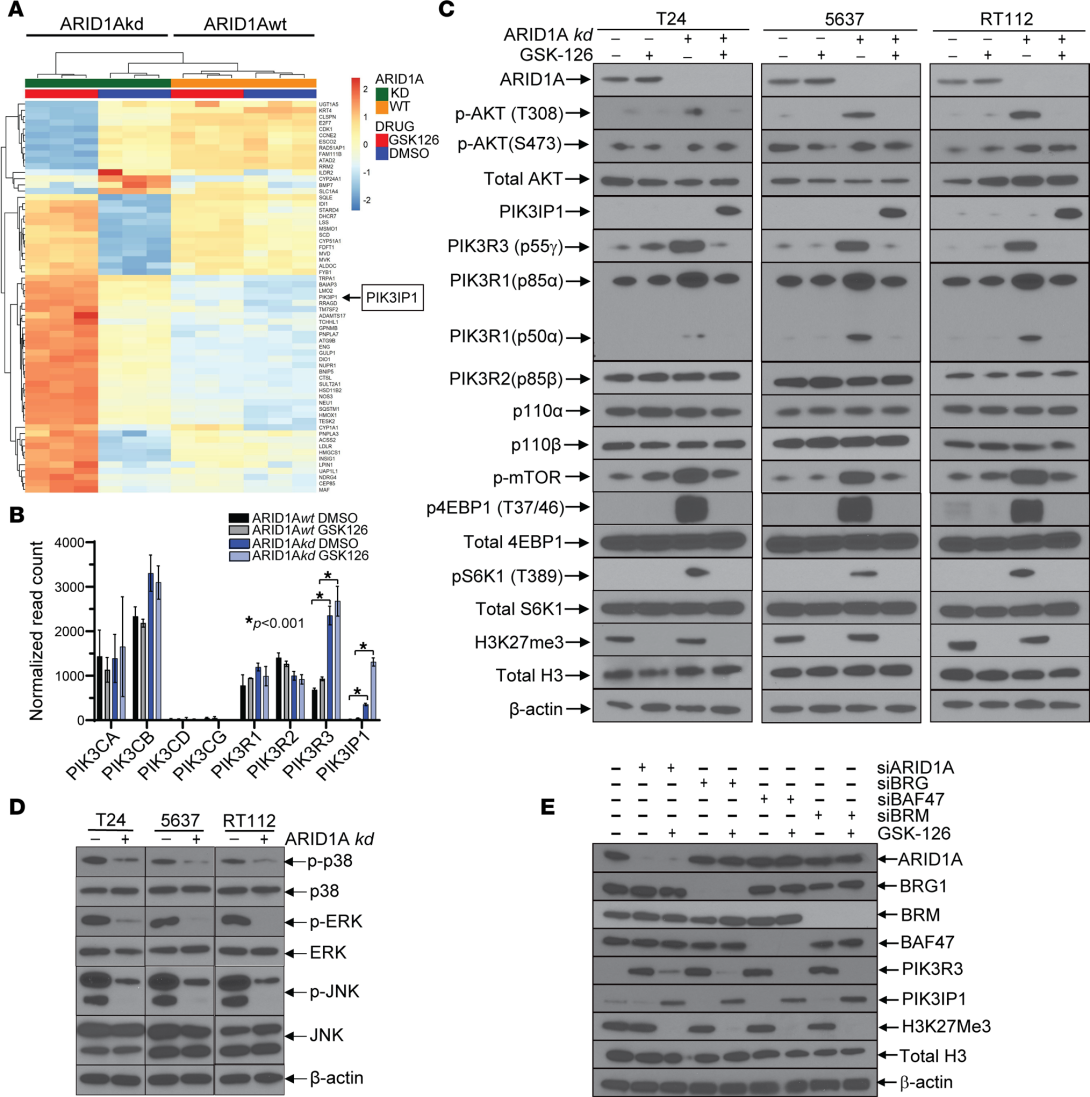
ARID1A deficiency leads to upregulation of PI3K signaling and downregulation of MAPK signaling, which results in a dependency on PI3K signaling. This dependency is targeted by GSK-126–mediated upregulation of the endogenous PI3K inhibitor, PIK3IP1. (**A**) Dendogram from whole transcriptomic RNA-Seq analysis showing differentially expressed genes between RT112 ARID1A*wt* cells treated with GSK-126 (5 μM for 24 hours) and RT112 ARID1A*kd* cells treated with GSK-126. PIK3IP1 is a putative tumor suppressor, an inhibitor of PI3K signaling, and a candidate for causing GSK-126 sensitivity in ARID1A*def* cells. (**B**) RNA-Seq subgroup analysis of major PI3K catalytic and regulatory subunits showing that PIK3R3 is upregulated in ARID1A*kd* cells. *t* tests were performed. (**C**) Immunoblot of the major PI3K/AKT/mTOR signaling cascade constituents showing that PIK3R3/p55γ (and to a lesser extent PIK3R1/p85α/p50α) is upregulated in ARID1A*kd* cells and corresponds with activation of AKT and downstream mTOR targets, p4EBP1 and pS6K1. These changes are abrogated upon treatment with GSK-126 (5 μM for 48 hours), which correlates with upregulation of PIK3IP1. (**D**) Immunoblots of ARID1A*kd* and ARID1A*wt* cells revealing downregulation of MAPK signaling in ARID1A*kd* cells, including p38, ERK, and JNK. (**E**) Immunoblot of RT112 cells with siRNA-mediated knockdown of various SWI/SNF (BAF) components, as indicated, with and without GSK-126 treatment shows that an intact complex is necessary to inhibit PIK3R3 expression and prevent GSK-126–mediated upregulation of PIK3IP1.

We hypothesized that ARID1A-deficient cells are more sensitive to GSK-126 due to transcriptional upregulation of specific tumor suppressors that remain transcriptionally repressed in ARID1A*wt* cells. To test this hypothesis, we performed RNA-Seq whole-transcriptomic analysis, comparing ARID1A*wt* and ARID1A*kd* RT112 bladder cancer cells with GSK-126 treatment or control. We isolated RNA after 24 hours to capture the early/primary transcriptional effects of GSK-126 while avoiding secondary effects due to lower viability and apoptosis initiation at later time points. Although there was a large number (>1,000) of differentially expressed genes when comparing untreated ARID1A*wt* cells with ARID1A*kd* cells at baseline (as expected given the function of ARID1A as a master transcriptional regulator), there were comparatively fewer (<70) differentially expressed genes in response to GSK-126 treatment. We specifically evaluated differentially expressed genes in GSK-126–treated ARID1A*kd* cells that were not differentially expressed following treatment of ARID1A*wt* cells (Figure 3A). Of these genes, one tumor suppressor, *PIK3IP1*, was particularly notable, as it attenuates PI3K/AKT/mTOR signaling through a direct inhibition of *PIK3CA*/p110α (37). This raised the hypothesis that ARID1A-deficient cells are dependent on PI3K signaling for survival, perhaps through an upregulation of PI3K constituents. To this end, we investigated the transcriptional levels of all of the class I PI3K subunits using the RNA-Seq data and found that ARID1A*kd* leads to upregulation of *PIK3R3*, a relatively uncharacterized regulatory subunit of PI3K that forms heterodimers with PIK3CA and modulates its activity (Figure 3B). We confirmed the RNA-Seq data via qRT-PCR and immunoblots and showed that ARID1A*kd* results in activation of the PI3K/AKT/mTOR pathway (as evidenced by higher levels of pAKT [Thr 308], phospho-mTOR, p4EBP1, and pS6K) and that GSK-126 treatment in ARID1A*kd* cells prevents this activation, which correlates with PIK3IP1 upregulation (Figure 3C). Furthermore, PIK3IP1 upregulation correlates with downregulation of *PIK3R3*/p55γ and *PIK3R1*/p85/p50α (Figure 3C) without a significant change in mRNA levels (Supplemental Figure 5, A and B), suggesting a posttranslational mechanism. Protein levels of other class I PIK3 subunits (p110α, p110β, and p85β) were relatively unchanged (Figure 3C). To determine if ARID1A is present at the promoters of *PIK3R3* and *PIK3IP1*, we performed cleavage under targets and release using nuclease (CUT&RUN) using 2 independent ARID1A antibodies and confirmed that ARID1A is present at the promoters of both *PIK3R3* and *PIK3IP1* (Supplemental Figure 5, C and D)

We hypothesized that ARID1A*kd* cells downregulate other proproliferation pathways to become dependent on PI3K/AKT/mTOR signaling for survival. This hypothesis was based on the following findings: (a) PI3K/AKT/mTOR signaling was increased in ARID1A*kd* cells (Figure 3C), but proliferation of ARID1A*kd* cells was no different from that of WT cells (Figure 2C). (b) GSK-126 treatment resulted in decreased viability of ARID1A*kd* cells (Figure 2C) despite decreasing pAKT levels only to levels found in ARID1A*wt* cells (which are resistant to GSK-126) (Figure 3C). We thus empirically compared the relative activation of proproliferation pathways prominent in bladder cancer biology and found that pERK, pJNK, and phospho-p38 were all lower in ARID1A*kd* cells (Figure 3D).

To test whether the intact SWI-SNF complex (also known as BAF) was necessary to prevent GSK-126 sensitivity, we knocked down key SWI-SNF components (BRG, BAF47, and BRM) via siRNA in RT112 cells. Knockdown of each component was associated with upregulation of PIK3R3 at baseline and upregulation of PIK3IP1 after treatment with GSK-126 (Figure 3E).

**Figure 4:**
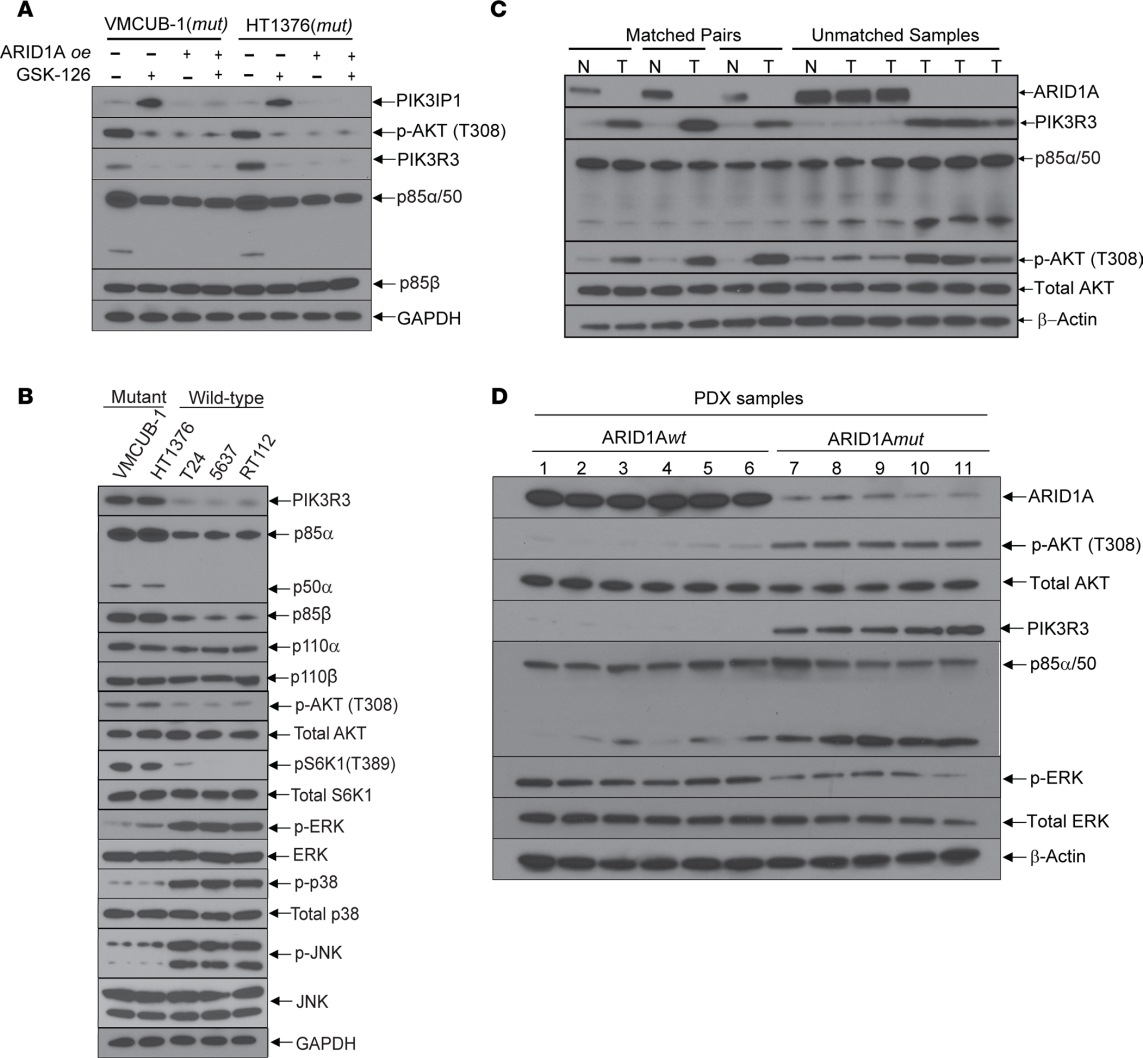
ARID1A deficiency correlates with upregulation of PIK3R3, activation of the PI3K/AKT signaling cascade, and downregulation of MAPK signaling in human bladder cancers. (**A**) Immunoblot of ARID1A-overexpressing (ARID1A*oe)* cells with or without GSK-126 (5 μM for 48 hours), showing that PIK3IP1 upregulation with GSK-126 treatment is abrogated by ARID1A reconstitution. These blots are from the same experiment shown in Figure 2F. (**B**) Immunoblots of bladder cancer cells with or without ARID1A mutations, indicating that ARID1A deficiency correlates with PI3K upregulation and MAPK downregulation. (**C**) Immunoblots of lysates from bladder tumors (T) and matched adjacent normal tissue (N) (as well as unmatched samples) from patients undergoing cystectomy, showing that tissues deficient in ARID1A have high levels of PIK3R3 and activated AKT. (**D**) Immunoblots using lysates from PDX models with ARID1A*wt* and *mut* alleles indicate that ARID1A deficiency correlates with increased PIK3R3 and pAKT and decreased MAPK activation. Corresponding sample numbers are noted in Methods.

Next, we tested whether ARID1A reconstitution/overexpression in ARID1A*mut* cells prevented this process. In ARID1A*mut* cells, pAKT and PIK3R3 levels were high at baseline, and treatment with GSK-126 decreased these levels (which correlated with PIK3IP1 upregulation) (Figure 4A). As hypothesized, ARID1A reconstitution lowered PIK3R3 and pAKT levels at baseline and prevented PIK3IP1 upregulation upon GSK-126 treatment (Figure 4A). Furthermore, when comparing ARID1A*mut* and WT cells, ARID1A*mut* lines showed higher levels of pAKT, PIK3R3, PIK3R1/p85α/p50α, and p85β and lower MAPK activation, indicative of an intrinsic shift in progrowth signaling dependent on ARID1A status (Figure 4B). Next, we sought to determine whether ARID1A deficiency correlated with PIK3R3 upregulation and PI3K/AKT pathway activation in human bladder cancers. Using lysates of 3 matched pairs of human bladder cancers and adjacent normal tissue, as well as 6 unmatched samples, we determined that tumors deficient in ARID1A protein had elevated PIK3R3 and pAKT levels (Figure 4C). Furthermore, using lysates from PDX models harboring ARID1A*wt* or -*mut* alleles, we determined that ARID1A-mutant/deficient tumors had elevated PIK3R3 and pAKT levels and decreased pERK levels (Figure 4D). These findings suggest that, in bladder cancer cells, ARID1A deficiency induces a biologic dependency on PI3K/AKT signaling that can be pharmacologically targeted through EZH2 inhibition and upregulation of PIK3IP1.

### PIK3IP1 upregulation is necessary and sufficient for GSK-126–mediated cell death in ARID1A-deficient bladder cancer cells.

**Figure 5:**
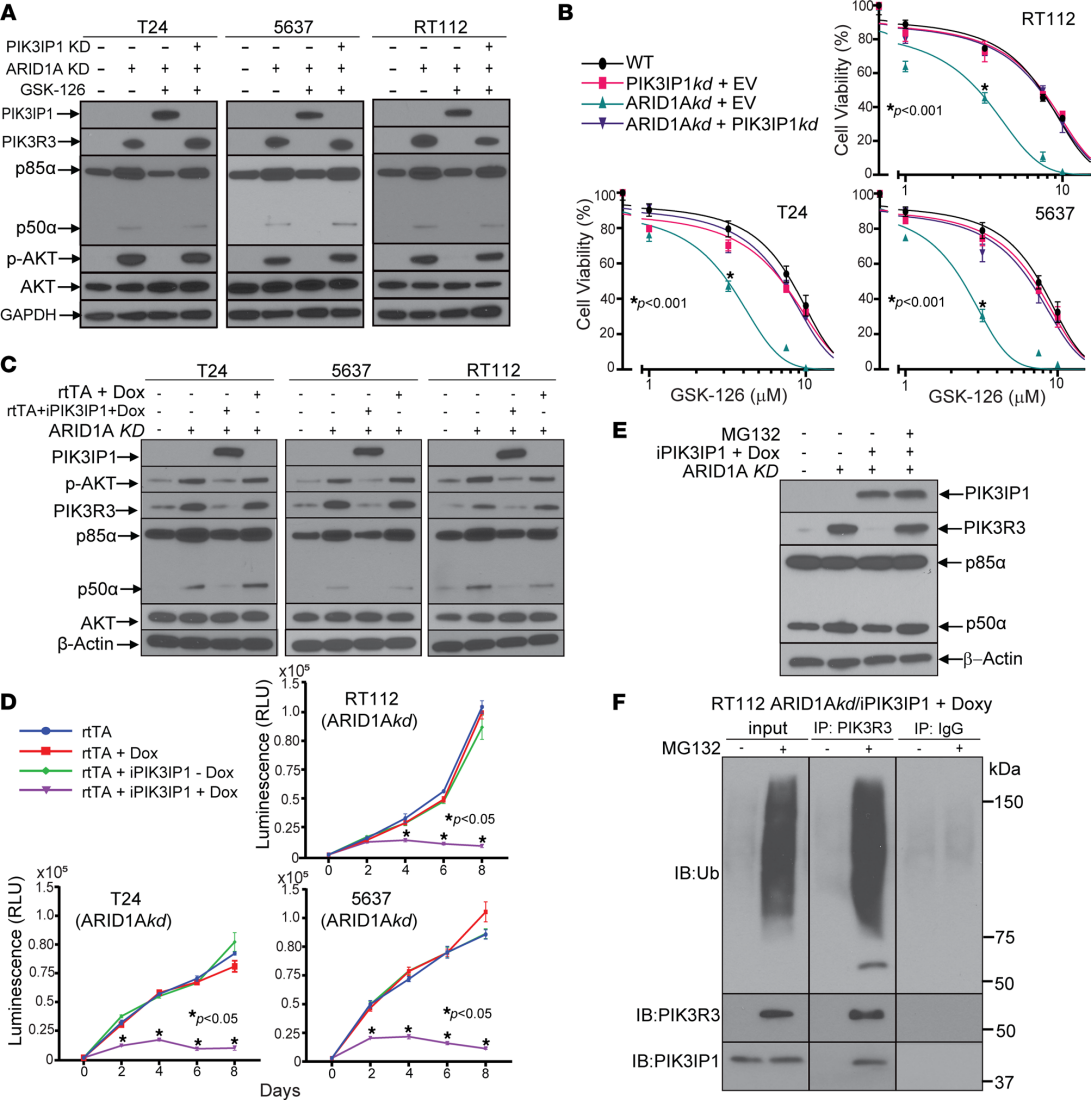
PIK3IP1 is necessary and sufficient for decreased viability of ARID1A*kd* cells and functions by inducing proteasomal degradation of PIK3R3. (**A**) Immunoblot analysis of PI3K constituents in ARID1A*kd*/PIK3IP1*kd* cells versus ARID1A*kd*/empty vector cells, showing that PIK3IP1 upregulation upon GSK-126 treatment (5 μM for 48 hours) is necessary to downregulate PIK3R3 and AKT activation. (**B**) Cell viability dose-response analysis with GSK-126 6-day treatment of ARID1A*kd*/PIK3IP1*kd* cells or empty vector (EV) controls, showing that PIK3IP1*kd* is sufficient to rescue the GSK-126 sensitivity phenotype in ARID1A*kd* cells. *t* test using IC_50_ values was performed. (**C**) Immunoblot analysis of ARID1A*kd* cell lines stably transfected with doxycycline-inducible PIK3IP1 (iPIK3IP1), showing that PIK3IP1 overexpression results in downregulation of PIK3R3 and AKT activation. rtTA denotes vector-encoding doxycycline-inducible transcriptional modulators (without inducible PIK3IP1). (**D**) Cell proliferation time course using the ARID1A*kd* cell lines in **C**, showing that PIK3IP1 overexpression results in less proliferation. *t* tests were performed. (**E**) Cotreatment of ARID1A*kd*/PIK3IP1 doxycycline-inducible cells with doxycycline and the proteasome inhibitor MG132 (15 μM for 12 hours) prevented PIK3R3 downregulation upon PIK3IP1 overexpression. (**F**) Cotreatment of ARID1Akd/iPIK3IP1 cells with doxycycline and MG-132, followed by immunoprecipitation of PIK3R3 resulted in coimmunoprecipitation of PIK3IP1 and poly-ubiquitination signal.

To determine if PIK3IP1 upregulation is necessary and sufficient for GSK-126 sensitivity in ARID1A-deficient bladder cancer cells, we generated an array of dual ARID1A/PIK3IP1 stable knockdown cells and ARID1A*kd*/PIK3IP1 stable overexpression cells (Figure 5, A and C). Stable knockdown of PIK3IP1 in ARID1A*kd* cells prevented GSK-126–mediated downregulation of PIK3R3 and pAKT and rescued/reversed the sensitivity to GSK-126, compared with empty vector (Figure 5B). We hypothesized that stable overexpression of PIK3IP1 in ARID1A*kd* cells would result in cell death, so we generated overexpression systems using a 2-vector doxycycline-inducible system. Overexpression of PIK3IP1 in ARID1A*kd* cells was sufficient to downregulate PIK3R3 and pAKT levels (Figure 5C), and it completely blocked cell growth (Figure 5D). As the downregulation of PIK3R3 upon PIK3IP1 overexpression was not associated with any changes in transcript levels (Figure 3 and Supplemental Figure 5A), we hypothesized that this was due to ubiquitin-mediated proteasome degradation. To test this, we treated ARID1A*kd*/PIK3IP1-inducible RT112 cells with doxycycline and the proteasome inhibitor MG-132 and found that proteasomal inhibition prevented PIK3IP1-dependent downregulation of PIK3R3 protein (Figure 5E). Finally, using ARID1A*kd*/PIK3IP1-inducible RT112 cells that had been treated with doxycycline and MG-132, we found that PIK3R3 immunoprecipitates contained PIK3IP1 and poly-ubiquitin moieties (Figure 5F). Together, these data suggest that PIK3IP1 binds to PIK3R3 and promotes poly-ubiquitination and degradation of PIK3R3.

### ARID1A deficiency correlates with sensitivity to PI3K inhibitors, which are synergistic with EZH2 inhibitors.

**Figure 6:**
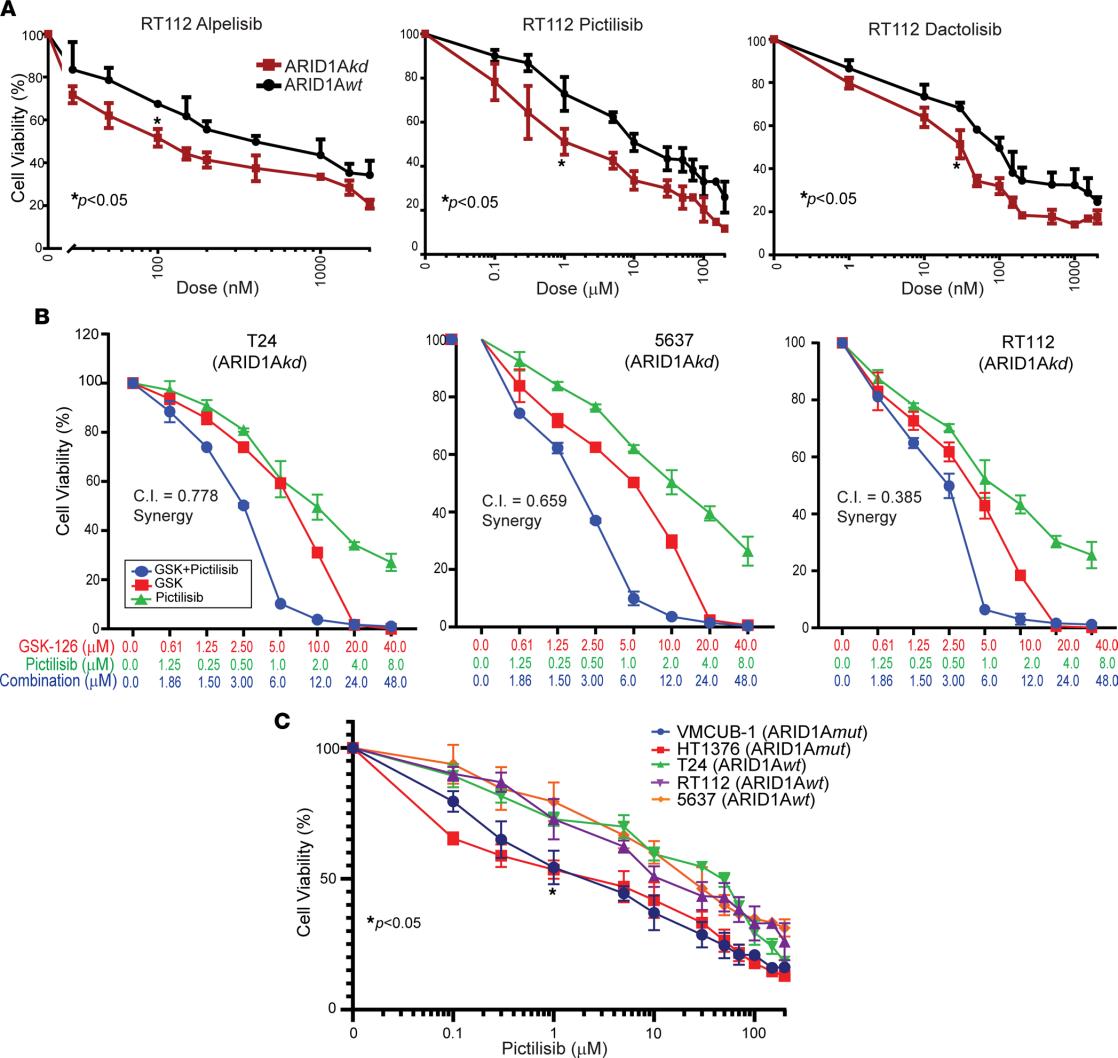
ARID1A deficiency sensitizes bladder cancer cells to PI3K inhibitors, which act synergistically with GSK-126. (**A**) Cell viability dose-response analysis of RT112 cells with ARID1A*kd* or empty vector (ARID1A*wt*) treated with alpelisib (a PI3K α-selective inhibitor), pictilisib (a PI3K class I selective inhibitor), or dactolisib (a dual PI3K/mTOR inhibitor) (all 48 hours). *t* tests of IC_50_ values were performed. (**B**) Dose-response synergy analyses were performed with GSK-126 and pictilisib using ARID1A*kd* cell lines (48 hours). Combination indices (C.I.) and synergism were calculated using the Chou-Talalay method. (**C**) Dose-response cell viability curves of bladder cancer cells with ARID1A WT and mutant alleles (48 hours treatment). Two-way ANOVA was performed using IC_50_ values.

To assess whether ARID1A deficiency sensitizes bladder cancer cells to PI3K inhibitors, we performed dose-response cell viability experiments comparing ARID1A*wt* and ARID1A*kd* cell lines in the presence of alpelisib (a PI3K α-selective inhibitor), pictilisib (a PI3K pan-class I inhibitor), or dactolisib (a dual PI3K/mTOR inhibitor). For all inhibitors tested, ARID1A*kd* cells were more sensitive than ARID1A*wt* cells (Figure 6A and Supplemental Figure 6, A–C). Because combination therapies are often utilized to maximize therapeutic benefit at lower doses and to avoid off-target side effects at higher doses, we tested the synergy of GSK-126 and pictilisib using a dose-response cell viability assay with 3 different ARID1A*kd* cell lines and found that each demonstrated synergy (based on combination indices < 0.85, calculated by the Chou-Talalay method) (Figure 6B and Supplemental Figure 6D). ARID1A*mut* cells were more sensitive to pictilisib than ARID1A*wt* cells (Figure 6C). These data suggest that ARID1A-deficient bladder cancers can be therapeutically targeted with EZH2 or PI3K inhibitors alone or ideally in combination.

### PIK3R3 upregulation is necessary and sufficient for PI3K/AKT pathway activation and increased bladder cancer cell proliferation.

**Figure 7:**
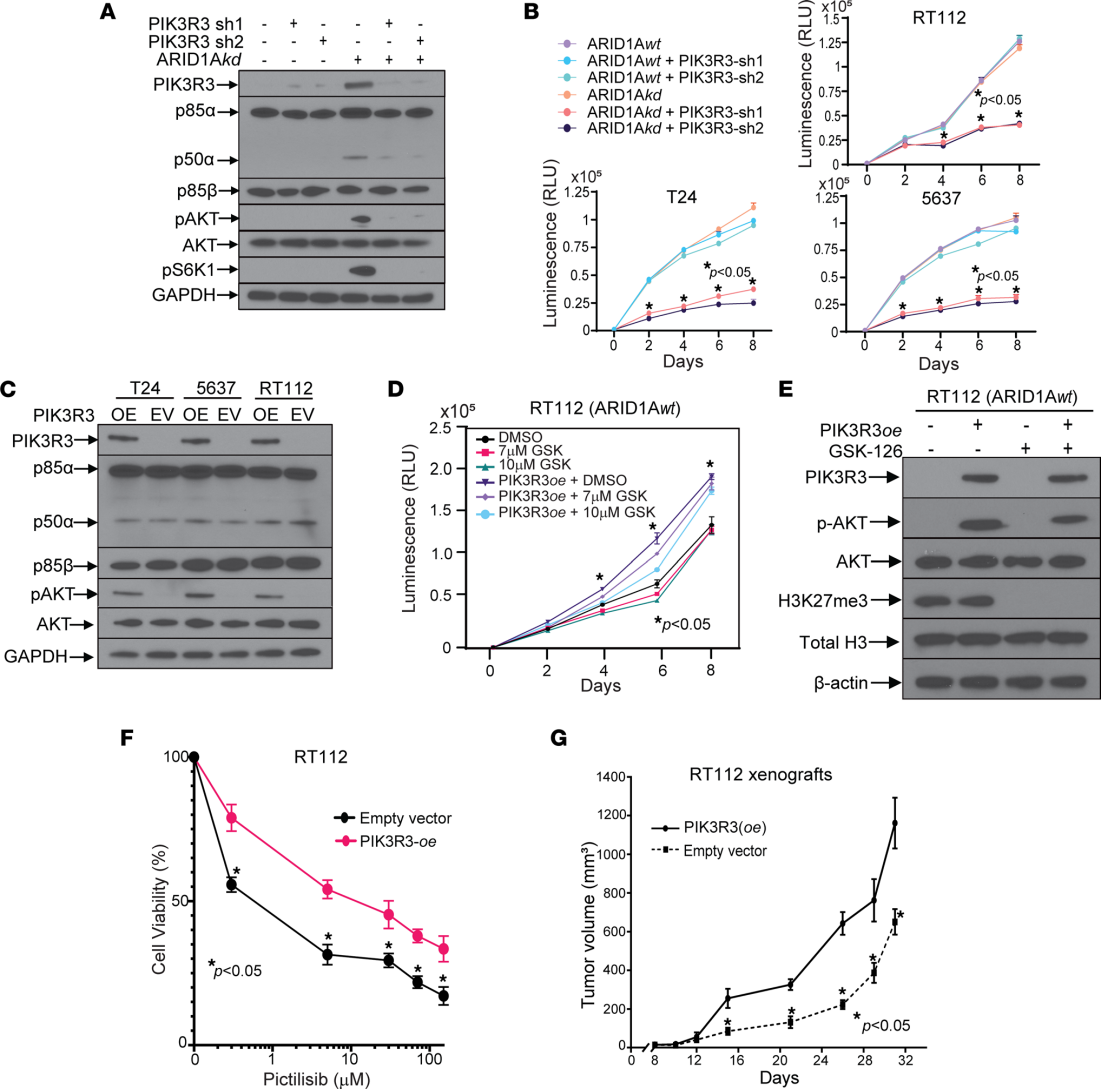
PIK3R3 upregulation in ARID1A*kd* cells is necessary and sufficient for bladder cancer cell proliferation, but it is not sufficient to convey GSK-126 or PI3K inhibitor sensitivity. (**A**) Immunoblots of lysates of RT112 ARID1A*kd* cells with PIK3R3 shRNA constructs (or empty vector) indicating that PIK3R3 KD prevents AKT and mTOR activation. (**B**) PIK3R3 knockdown inhibited proliferation of ARID1A*kd* cells but not ARID1A*wt* cells. *t* tests were performed. (**C**) PIK3R3 overexpression (OE) in ARID1A*wt* cells increased AKT activation compared with cells transfected with an empty vector (EV). (**D**) RT112 cells with PIK3R3 overexpression show increased proliferation at baseline but no increased sensitivity to GSK-126. Two-way ANOVA was performed. (**E**) Lysates from **D**, indicating that GSK-126 (5 μM for 48 hours) inhibits H3K27 trimethylation in these cells. (**F**) Dose-response assay using RT112 ARID1A*wt* cells with PIK3R3 overexpression or empty vector showing that PIK3R3 overexpression causes resistance to the PI3K inhibitor, pictilisib (48 hours). *t* tests were performed. (**G**) Xenografts of RT112 ARID1A*wt* cells with PIK3R3 overexpression grow faster than cells transfected with empty vector controls. *t* tests were performed.

To determine whether PIK3R3 upregulation is necessary and sufficient for PI3K/AKT pathway activation and rapid cell proliferation, we generated dual ARID1A/PIK3R3 stable knockdown cells and ARID1A*wt* cells stably overexpressing PIK3R3 along with empty vector controls (Figure 7). Knockdown of PIK3R3 decreased pAKT and pS6k levels in ARID1A*kd* cells (Figure 7A) and inhibited growth of ARID1A*kd* cells but not ARID1A*wt* cells (Figure 7B). Stable overexpression of PIK3R3 in ARID1A*wt* cells was sufficient to increase pAKT levels (Figure 7C) and resulted in increased proliferation; however, these cells were not more sensitive to GSK-126 (Figure 7D and Supplemental Figure 7A). Immunoblot analysis confirmed the activity of GSK-126 in these cells, as evidenced by lower H3K27me3 levels (Figure 7E). Furthermore, PIK3R3 overexpression resulted in resistance to PI3K inhibitors (Figure 7F and Supplemental Figure 7B), and xenografts with PIK3R3 overexpression grew faster than xenografts transfected with empty vector (Figure 7G and Supplemental Figure 7C). Finally, utilizing the TCGA data set, we found that ARID1A*mut* tumors had higher transcript levels of PIK3R3 than ARID1A*wt* tumors (Supplemental Figure 7D).

Together, these data suggest that PIK3R3 upregulation in ARID1A*kd* cells drives activation of the PIK3/AKT/mTOR pathway, a process sufficient to increase proliferation in ARID1A*wt* cells. However, due to the lack of dependency of ARID1A*wt* cells on the PI3K/AKT pathway (as evidenced by intact ERK, JNK, and p38 signaling cascades), PIK3R3 upregulation alone does not result in pharmacologic vulnerabilities in the PI3K/AKT pathway.

## Discussion

Metastatic urothelial carcinoma is generally incurable, with modest survival benefit provided by cisplatin-based first-line chemotherapy (median survival, ~15 months) (38). Durable benefits with postplatinum PD-1/L1 inhibitors extend survival to a minority of patients (~20%), and the median survival is less than 1 year (39). Third-line salvage therapies (e.g., enfortumab vedotin, sacituzumab govitecan) are not curative but provide incremental benefits, with median overall survival of approximately 1 year. However, the first targeted agent for bladder cancer, erdafitinib, is active and is approved to treat postplatinum patients with activating somatic *FGFR2/3* mutations or fusions, which are seen in approximately 15% of patients (40). Hence, new therapeutic approaches are needed for treatment of patients with bladder cancer. These will be achieved with better understanding of therapeutically actionable targets and mechanisms of resistance. Given the heterogeneity of this malignancy with multiple genomic alterations, there remains a role for rational approaches targeting these subsets of patients.

*ARID1A* is frequently mutated across a wide variety of human cancers, including bladder, gastric, pancreatic, and ovarian cancers (6, 7). As ARID1A is the DNA-binding subunit of the large, approximately 1.15 MDa SWI/SNF multisubunit complex, its loss through nonsense mutations is thought to result in complex disassembly. Mutation of just 1 allele of *ARID1A* results in embryonic lethality for mice and contributes to tumorigenesis (8, 9). Thus, tightly controlled protein levels of ARID1A are critical for normal development and disease prevention.

Although loss of tumor suppressors such as ARID1A are difficult to target directly, oftentimes these losses result in therapeutic vulnerabilities that can be targeted through a synthetic lethality approach. For various bladder cancer cell lines with ARID1A-truncating mutations or shRNA-mediated depletion, our experiments revealed that EZH2 inhibition is synthetically lethal for bladder cancer cells with ARID1A deficiency. There are similar findings for ovarian clear cell carcinomas (OCCCs) (22). Other groups have investigated this relationship in bladder cancer cells and come to somewhat different conclusions (41). The cells in these studies, however, were treated with EZH2 inhibitor for only 2–3 days and showed no specific sensitivity for EZH2 inhibition, whereas we found that at least 6–8 days of treatment is necessary to see substantial differences in viability. This could explain the differences between these results.

Pharmacologic inhibitors of EZH2 are currently being investigated in a variety of tumor types, including lymphomas, sarcomas, and advanced treatment-resistant solid tumors (reviewed in refs. 20). B cell lymphomas often have activating mutations in EZH2, and some subtypes of sarcomas have mutations in SWI-SNF subunits *SMARCB1* (BAF47) or *SMARCA4* (BRG1). In fact, the EZH2 inhibitor tazemetostat was approved by the FDA in 2020 for the treatment of advanced epithelioid sarcoma (42). Thus, there is a rationale to repurpose EZH2 inhibitors for the pharmacologic treatment of patients with bladder cancers harboring somatic truncating mutations in *ARID1A*. A phase I/II trial is currently investigating the combination of tazemetostat/pembrolizumab in patients with molecularly unselected, advanced urothelial carcinoma (Clinicaltrials.gov NCT03854474) and another trial is investigating tazemetostat alone in advanced solid tumors harboring an ARID1A mutation (Clinicaltrials.gov NCT05023655). The results herein suggest that subgroup analyses of these and other EZH2 inhibitor trials should focus on patients with bladder cancer with ARID1A-deficient tumors.

We discovered that ARID1A mutations and/or deficiency leads to a dependency on PI3K/AKT/mTOR signaling via upregulation of PIK3R3 and downregulation of MAPK signaling. The PI3K/AKT/mTOR pathway is involved in bladder tumorigenesis, as a substantial proportion of these tumors have activating mutations in PIK3CA (2). Among the regulatory subunits, PIK3R3 is least characterized. Little is understood about how it preferentially interacts with the various catalytic subunits, how it competes with other regulatory subunits, and how heterodimers containing PIK3R3 function in the presence of PI3K pharmacologic inhibitors. To determine if PIK3R3 upregulation in ARIDA-deficient (ARID1A*def*) cells is specific to bladder cancer, we performed an in silico meta-analysis focused on the differential expression of PI3KR3 in all publicly available transcriptomic data sets comparing ARID1A-deficient and WT cells (see Supplemental Figure 8 for accession numbers). Using 6 separate data sets from 6 different cancer cell lines (including 1 OCCC), only 1 cell line (cholangiocarcinoma, HuCCT1) showed a statistically significant increase in PIK3R3 in ARID1A-deficient cells (Supplemental Figure 8), suggesting that the molecular mechanisms that lead to PIK3R3 upregulation in bladder cancer cells may be uncommon in other tumor types. Experiments are underway to determine these mechanisms. Downregulation of MAPK pathways in ARID1A-deficient endometrial carcinoma has been described previously (43), but whether this is universal among ARID1A-deficient tumors remains to be determined.

Inhibition of EZH2 in ARID1A*def* bladder cancer cells results in upregulation of PIK3IP1, an endogenous inhibitor of PI3K signaling. Although other data suggest that PIK3IP1 functions through direct binding and inhibition of PI3K catalytic subunit activity (37, 44), we revealed what we believe to be a novel mechanism by which PIK3IP1 expression induces proteasomal degradation of the regulatory subunit PIK3R3. In OCCCs, ARID1A and EZH2 compete (along with histone deacetylase 2) to modulate expression of the *PIK3IP1* gene (22, 25). However, while PIK3IP1 protein levels are increased in response to GSK-126 in ARID1A*def* cells in both OCCC and bladder cancer, the effects of ARID1A levels on PIK3IP1 expression are opposite between the cell types. Factors contributing to this difference and the ramifications of this difference are being investigated.

In conclusion, ARID1A-deficient bladder cancers are dependent on PI3K signaling, which can be pharmacologically targeted with EZH2 and/or PI3K inhibitors. Our results show a potentially novel role for PIK3R3 as an oncogene in bladder cancer biology. Finally, these data demonstrate that the role of PIK3IP1 in the sensitivity of ARID1A*def* cells to EZH2 inhibitors may be shared among multiple tumor types. In sum, clinical applications targeting these molecules in ARID1A-deficient bladder cancers should be pursued.

## Methods

Further information can be found in Supplemental Methods.

Experimental design

### Cell lines and reagents.

Bladder cancer cell lines HT1376 (RRID CVCL_1292), T24 (CVCL_0554), and 5637 (CVCL_0126) were obtained from ATCC, and RT112 (CVCL_1670) and VM-CUB1 (CVCL_1786) were obtained from DSMZ. Cell lines were authenticated by the vendors and tested for mycoplasma prior to use. These were grown in Dulbecco’s 90% MEM (4.5 g/L glucose) with penicillin/streptomycin (100 U/mL) and 10% fetal bovine serum (MilliporeSigma) in 5% CO_2_ incubators. GSK-126 (HY-13470), alpelisib (HY-15244), and dactolisib (HY-50673) were obtained from MedChem Express. Pictilisib (S1065), MG132 (S2619), MAK-683, EPZ-6438, and CPI-1205 were obtained from Selleckchem. Captisol (RC-0C7-020) was obtained from CyDex Pharmaceuticals.

### Cell proliferation and dose-response assays.

After exposure to drugs as indicated, cell viability was measured by luminescence using CellTiter-Glo 2.0 (Promega), according to the manufacturer’s instructions. Luminescence was measured on a Synergy HTX multimode reader (BioTek Instruments Inc.). For dose-response experiments, media/drug was replenished every other day. Percent viability of cells was calculated as follows: percentage viability = 100 − (treated relative luminescence unit/nontreated RLU)/100. For drug combination studies, data were analyzed for synergistic interactions using CompuSyn software and the Chou–Talalay method (45).

### RNA isolation and RNA-Seq analysis.

Total RNA from cultured cells was extracted with Direct-zol RNA miniprep kits (Zymo Research). Library preparation was performed with purified, extracted RNA using KAPA Stranded mRNA-seq kits (Kapa) according to the manufacturer’s instructions. Twelve samples with various adapters were pooled to create a 15 nM multiplexed sample for sequencing. This pooled sample was diluted to 1.65 pM and spiked with 1% PhiX bacterial genome as a positive control for alignment. High-throughput sequencing with 75 bp single-end reads was performed on an Illumina NextSeq 550 using an Illumina NextSeq 500/550 High Output Kit. Reads were aligned to the human transcriptome GENCODE v32 (GRCh38.p13) using STAR and counted using Salmon. Normalization and differential expression analysis were performed using the R package DESeq2. Genes for which there were fewer than 3 samples with normalized counts equal to or less than 5 were filtered out of the final data set. A Benjamini-Hochberg-adjusted *P* value of less than 0.05 and a log_2_ fold change of 1 were thresholds used to identify differentially expressed genes between treatment conditions. Conditions were run in biologic triplicates.

### Xenograft tumor growth assays.

For tumor xenograft experiments, *NU/J nu/nu* male and female mice aged 6–8 weeks (*n* = 5 for each group) from The Jackson Laboratory (RRID IMSR_JAX:002019) were injected subcutaneously into the right dorsal flanks with human bladder cancer cells (1 × 10^6^ to 2 × 10^6^ cells in 50 μL of incomplete media without FBS and 50 μL Matrigel, 354277, Corning). Tumor growth was measured with Vernier calipers and recorded weekly. Tumor volume was calculated with the formula: 0.5 × tumor length × tumor width^2^. Animals were randomized prior to treatment initiation. GSK-126 was administered intraperitoneally at a dose of 100 mg/kg daily after tumor volume reached 150 to 200 mm^3^. The final volume of drug/vehicle was 0.2 mL per 20g body weight in 20% Captisol, adjusted to a pH of 4–4.5 with 1 N acetic acid. Mouse body weights were monitored to assess toxicity. At the end of the treatment tumors were excised, weighed, processed, and stored for downstream molecular analysis. Statistical analyses were conducted by 2-tailed Student’s *t* tests.

### Xenografts derived from patients with bladder cancer.

Bladder cancer PDX models were provided by The Jackson Laboratory. PDXs were developed through subcutaneous implantation of clinical tumor tissues into immunodeficient NOD.Cg-*Prkdcscid Il2rgtm1Wjl*/SzJ (NSG; RRID:IMSR_JAX:005557) female mice, followed by serial passaging as previously described (32). All experiments utilized PDX models within the first 5 passages.

### PDX bladder cancer mouse models.

Six- to eight-week-old female beige SCID mice bearing bladder cancer PDXs (University of California, Davis, ID BL0293, or The Jackson Laboratory, model TM00016; University of California, Davis, ID BL0269, or The Jackson Laboratory, model TM00015) were utilized. Fresh PDX specimens (3–5 mm^3^) were implanted subcutaneously into the flanks of SCID mice. Experimental design followed the xenograft protocol as above.

For immunoblot analysis, various PDX tissues from tumors harboring *ARID1A* WT or mutant alleles were purchased from The Jackson Laboratory. ARID1A*wt* PDX included TM00015 (sample 1 in Figure 4D), TM00024 (sample 2), TM00013 (sample 3), TM00020 (sample 4), J000107333 (sample 5), and TM01029 (sample 6). ARID1A*mut* PDX included TM00016 (sample 7), J000109799 (sample 8), J000108112 (sample 9), J000102326 (sample 10), and J000100646 (sample 11).

### Immunoblot analyses.

Antibodies, listed in Supplemental Table 1, were used at dilutions optimized in our laboratory. For immunoblot analysis, protein samples were separated by sodium dodecyl sulfate–polyacrylamide gel electrophoresis and transferred onto Immobilon-P PVDF membranes (EMD Millipore). The membranes were incubated for 1 hour in blocking buffer (Tris-buffered saline, 0.1% Tween [TBS-T], 5% nonfat dry milk), followed by incubation overnight at 4°C with the primary antibody. After a wash with TBS-T, the blots were incubated with a horseradish peroxidase–conjugated secondary antibody, and signals were visualized by Luminata Crescendo chemiluminescence Western blotting substrate as per the manufacturer’s protocol (EMD Millipore). See complete unedited blots in the supplemental material.

### Immunoprecipitation.

Cell lysate (400 μg) was incubated with either anti-PIK3R3 or IgG control (5 μg) overnight at 4°C with rotation. Then, 25 μL of washed Protein A/G magnetic beads (Thermo Fisher Scientific) was added and incubated 1–2 hours at 4° with rotation. The bead/immune complex was then washed 3 times with 20 mM Tris-HCl, 150 mM NaCl, 2 mM EDTA (pH 8.0). The immunoprecipitated proteins were eluted by boiling beads in the Laemmli buffer and analyzed by immunoblot analysis.

### Preparation of human bladder cancer lysates.

As previously described (46), after pathologic stage and grade determination, protein lysates were prepared from human bladder cancer samples and surrounding normal mucosa, using tissues from radical cystectomies.

### RNA interference and transfection.

As described previously (47), siRNA duplexes (listed in Supplemental Table 2) were purchased from Dharmacon/Horizon Discovery. Transfections were performed with Lipofectamine RNAiMAX (Life Technologies) reagent per the manufacturer’s instructions. Seventy-two hours after the transfection, cells were harvested for immunoblot analysis.

### ARID1A shRNA lentivirus production.

High-titer lentivirus was generated following a protocol previously described in detail (48). In brief, pMD2.G (Addgene, 12259), psPAX2 (Addgene, 12260), and pLKO.1-shARID1A (MISSION shRNA; MilliporeSigma), including either TRCN0000059089 (sh1) or TRCN0000059090 (sh2) were used. ARID1Ash2 was used in experiments where one shRNA was utilized. For negative control pLKO.1 GFP shRNA (Addgene, 30323) was used. These lentiviruses were used to infect ARID1A WT bladder cancer cells, which were then selected for stably transfected clones using puromycin (Gibco, Thermo Fisher Scientific) at a concentration of 1–2 μg/mL for 2 weeks. Stable clones were selected and tested for ARID1A knockdown efficiency via immunoblots.

### Generation of ARID1A-expressing lentiviruses.

*ARID1A* cDNA-expressing lentiviruses were constructed using the protocol described above. pLenti-puro-*ARID1A* (Addgene, 39478) or empty vector–containing (p-Lenti-puro, Addgene, 39481) lentiviruses were used to infect *ARID1A* mutant bladder cancer cells, which were then selected for stably transfected clones using puromycin (Gibco, Thermo Fisher Scientific) at a concentration of 1–2 μg/mL for 2 weeks. Stable clones were selected and tested for ARID1A overexpression via immunoblots.

### Plasmids and antibodies.

A human PIK3IP1 stable overexpression system was custom-designed and purchased from VectorBuilder. This is a 2-vector system, with 1 vector expressing doxycycline-inducible transcriptional modulators (pLV[Exp]- CMV>tTS/rtTA/Hygro) and a second vector containing h*PIK3IP1* downstream from an inducible promoter (pLV[Exp]-Neo-TRE> *hPIK3IP1*[NM_052880.5]). *PIK3R3* overexpression vectors were custom-designed and purchased from Vector Builder (pRP[Exp]-Neo-CMV>*hPIK3R3*[NM_003629.4], along with an empty vector control (pRP[Exp]-Neo-CMV>ORF_Stuffer). PIK3R3 shRNA-containing lentivirus was purchased from MilliporeSigma (TRCN0000195671 [sh1] and TRCN0000033290 [sh2]), with pLKO.1-CMV-Neo vector as empty vector control. PIK3R3 sh1 was used in experiments where 1 shRNA is indicated. PIK3IP1 shRNA–containing lentivirus was purchased from MilliporeSigma (TRCN0000138560) with pLKO.1-CMV-Neo vector as empty vector control. The infected cells were selected with puromycin (1–2 μg/mL), neomycin (800–900 μg/mL), and hygromycin (150–200 μg/mL) as needed.

The antibodies used are listed in Supplemental Table 1. Of note, the PIK3R1 antibody was chosen given its epitope in the C-terminus and its capacity to detect all 3 splice variants of the gene, p85α/p55α/p50α. Using this antibody, we detected p85α and p50α but not p55α.

### Data availability.

The RNA-Seq data generated in this study are publicly available in Gene Expression Omnibus (GEO) at GSE183777.

### Statistics.

All results are representative of at least 3 biologic replicates, unless otherwise specified. All data points are shown as the mean ± SD and are representative of at least 3 technical replicates, unless otherwise specified. Two-way ANOVA or 2-tailed Student’s *t* tests were performed using GraphPad Prism v7.03 to determine significant differences between control and experimental groups, as mentioned in the figure legends. *P* values of less than 0.05 were considered statistically significant.

### Study approval.

Studies of human bladder cancer lysates were performed after IRB approval at the University of Alabama at Birmingham (X120917005) and obtaining written informed consent from patients. Animal studies were performed at University of Alabama at Birmingham and University of California, Davis, with approval of their Institutional Animal Care and Use Committees (protocol 19564 at University of California, Davis, and 21573 at University of Alabama at Birmingham).

## Author contributions

HR provided experimental design and interpretation, generated data, and prepared and reviewed the manuscript. HR was chosen as first listed co–first author because of his extensive data generation and manuscript preparation. DSC and CB provided experimental design and interpretation, generated data, and prepared and reviewed the manuscript. SN and SAHB provided experimental design and generated data. AKS, KRS, AHM, and TR generated data, provided experimental interpretation, and prepared and reviewed the manuscript. SA provided experimental interpretation and prepared and reviewed the manuscript. MLE and ADR generated data, provided experimental interpretation, and prepared and reviewed the manuscript. GN generated data and provided experimental interpretation. UM prepared and reviewed the manuscript. GJN provided experimental interpretation, prepared and reviewed the manuscript, and obtained project funding. CRM provided experimental interpretation and prepared and reviewed the manuscript. CP and GS provided experimental design and interpretation, prepared and reviewed the manuscript, and obtained project funding. SV and JEF provided experimental design and interpretation, obtained project funding, and prepared and reviewed the manuscript.

## Supplementary Material

Supplemental data

## Version 1 07/19/2022

In-Press Preview

## Version 2 08/22/2022

Electronic publication
