# Supplementary material for: ARID1A-deficient bladder cancer is dependent on PI3K signaling and sensitive to EZH2 and PI3K inhibitors
Source: JCI Insight. 2022 Aug 22;7(16):e155899. doi: 10.1172/jci.insight.155899 (PMC9462490; doi:10.1172/jci.insight.155899)

A

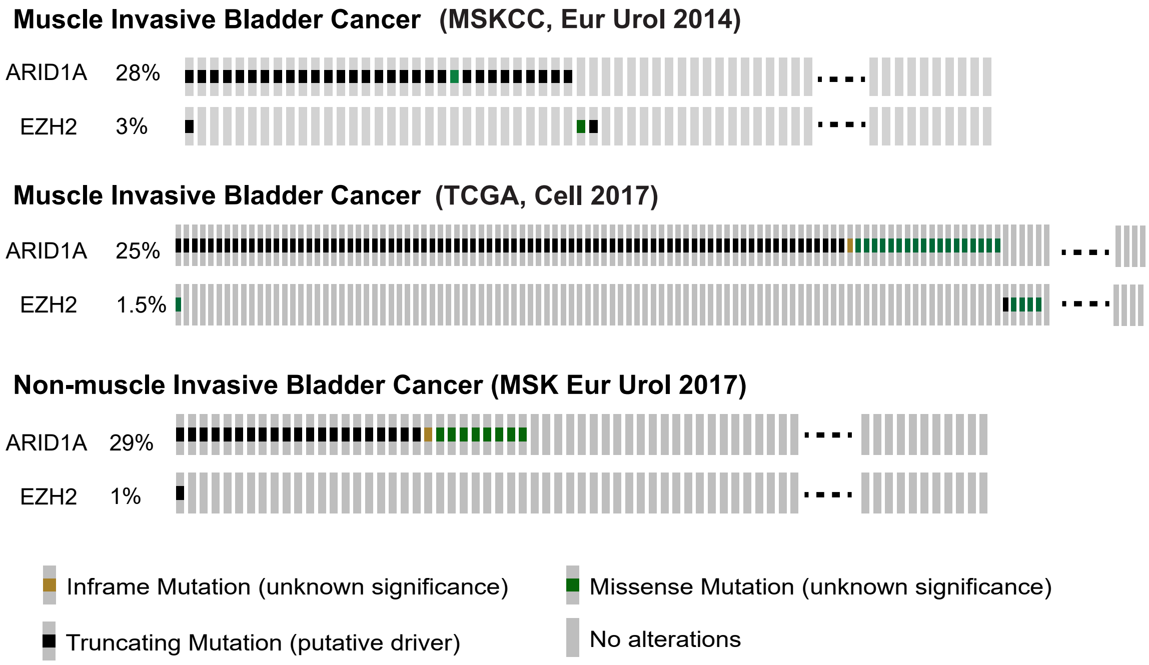

Muscle Invasive Bladder Cancer (MSKCC, Eur Urol 2014)
ARID1A 28%
EZH2 3%
Muscle Invasive Bladder Cancer (TCGA, Cell 2017)
ARID1A 25%
EZH2 1.5%
Non-muscle Invasive Bladder Cancer (MSK Eur Urol 2017)
ARID1A 29%
EZH2 1%
Inframe Mutation (unknown significance)
Missense Mutation (unknown significance)
Truncating Mutation (putative driver)
No alterations

B

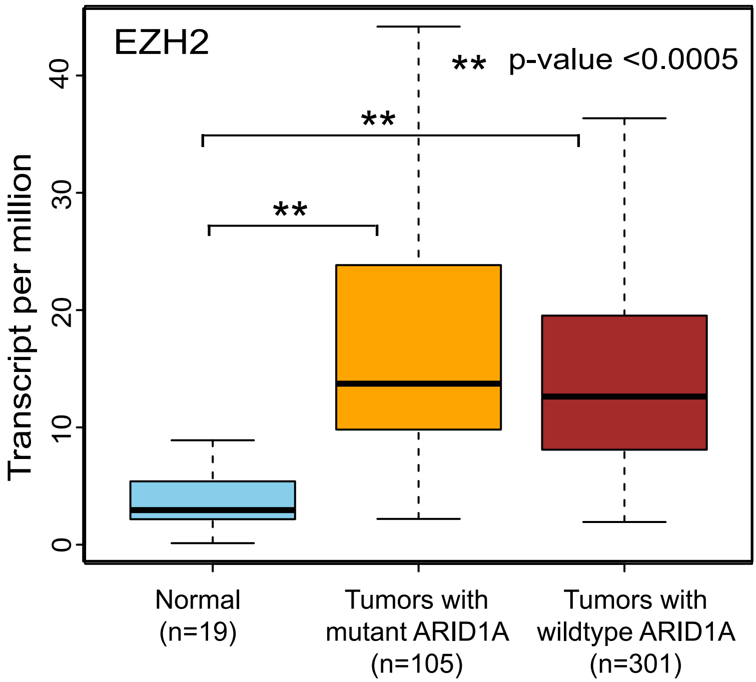

EZH2
** p-value <0.0005
**
**
Transcript per million
0
10
20
30
40
Normal (n=19)
Tumors with mutant ARID1A (n=105)
Tumors with wildtype ARID1A (n=301)

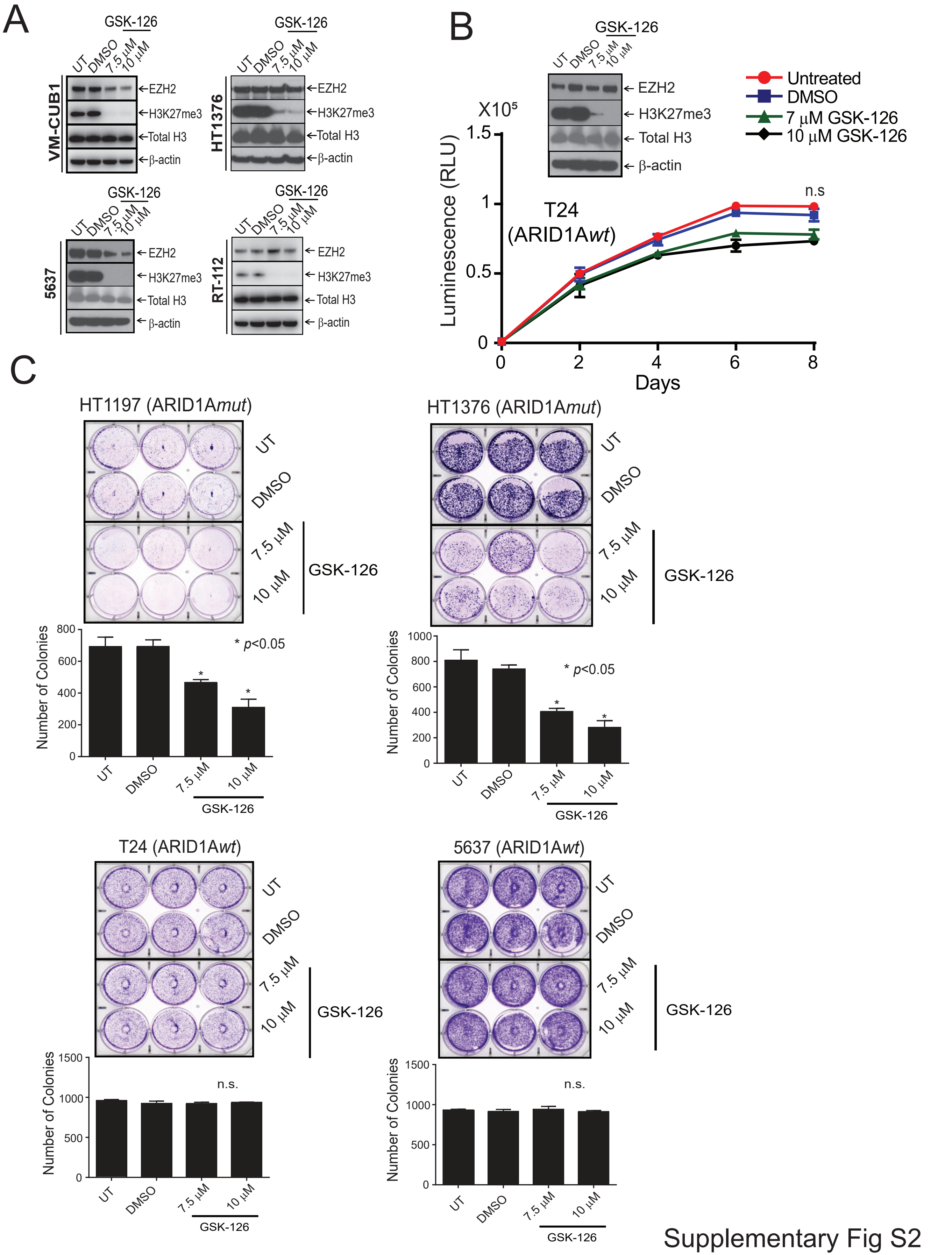

Supplementary Fig S2

Supplementary Fig S3

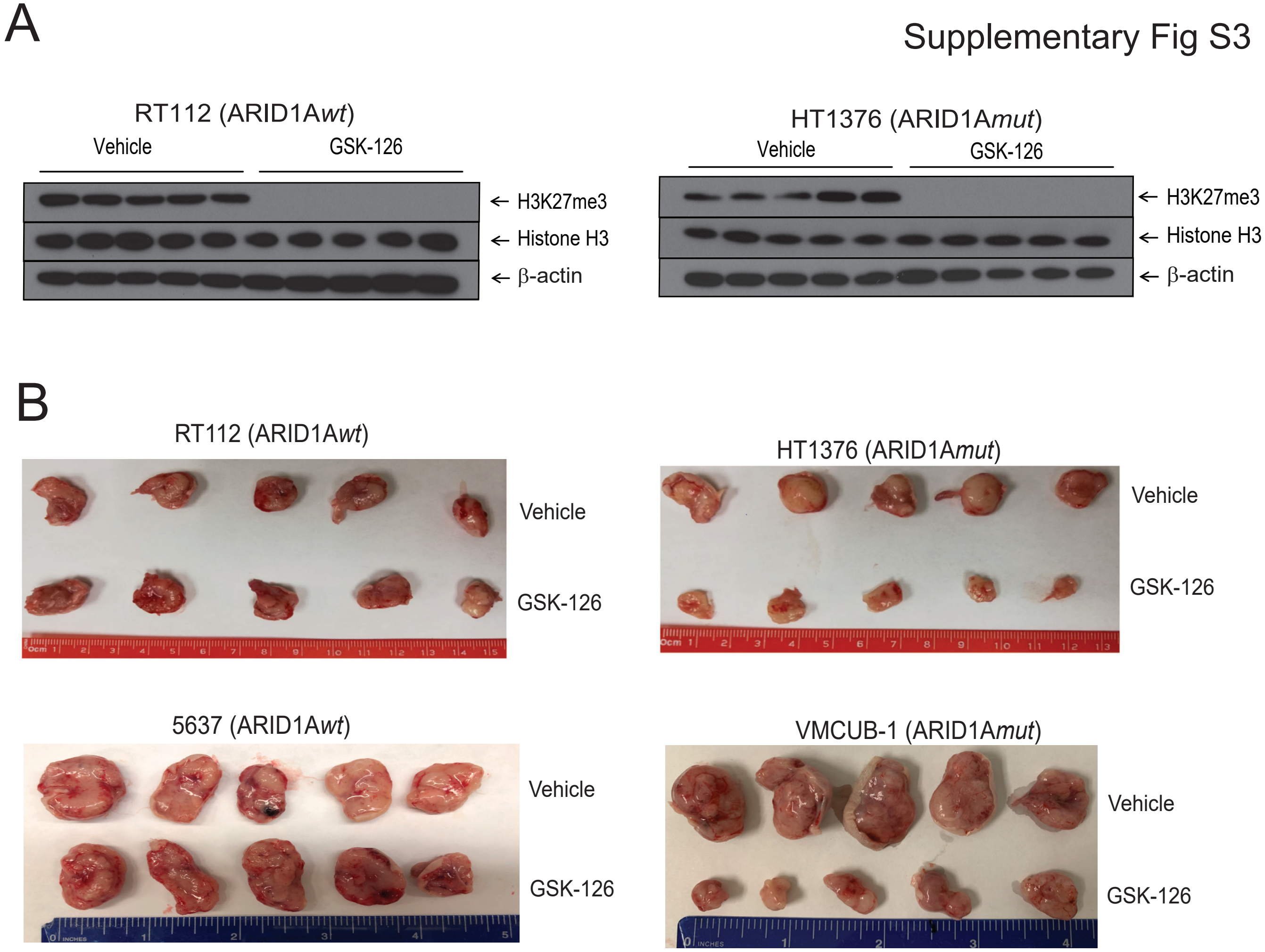

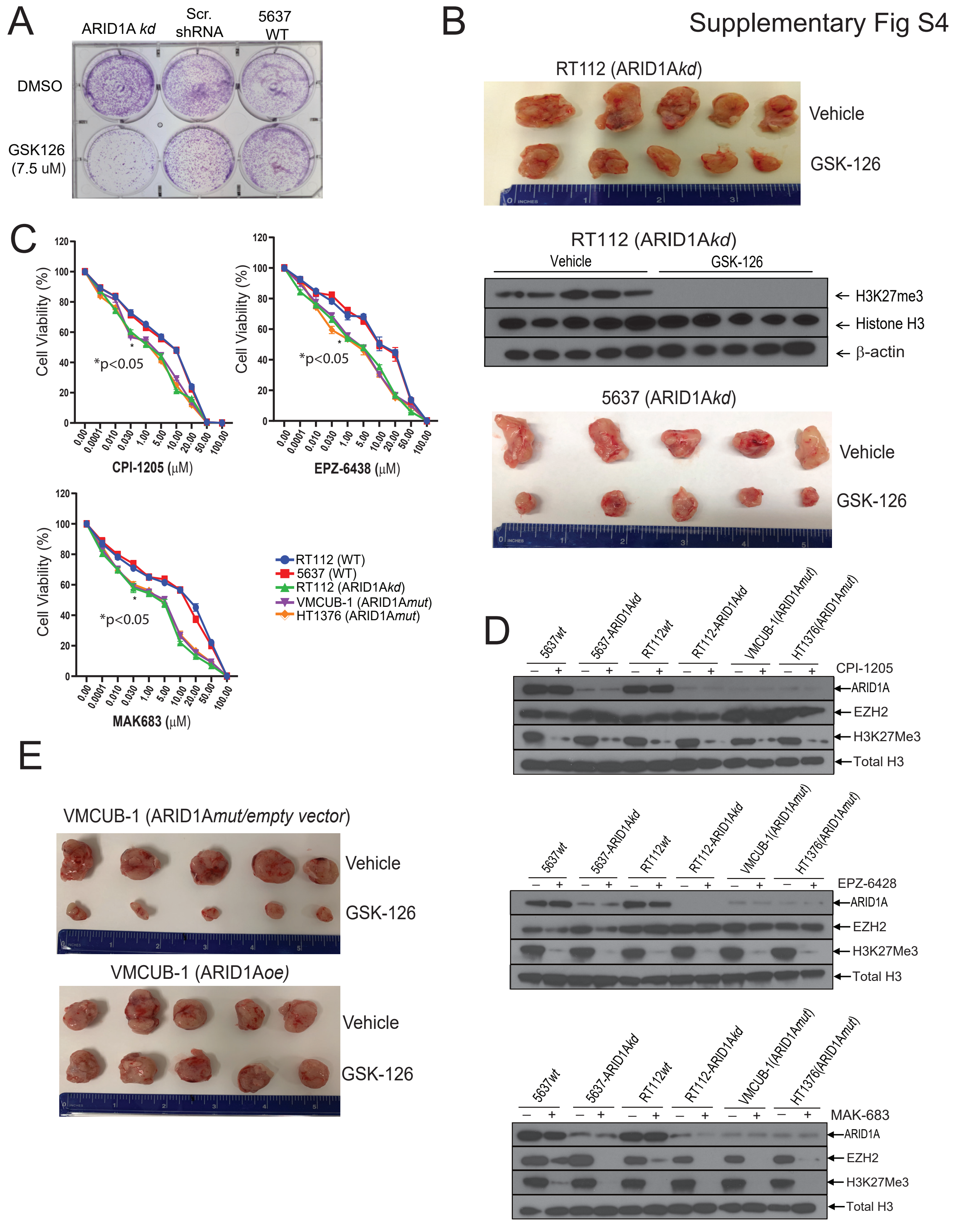

Supplementary Fig S4
A
ARID1A kd
Scr. shRNA
5637 WT
DMSO
GSK126 (7.5 uM)
B
RT112 (ARID1Akd)
Vehicle
GSK-126
RT112 (ARID1Akd)
Vehicle
GSK-126
H3K27me3
Histone H3
β-actin
5637 (ARID1Akd)
Vehicle
GSK-126
C
Cell Viability (%)
*p<0.05
CPI-1205 (μM)
EPZ-6438 (μM)
MAK683 (μM)
RT112 (WT)
5637 (WT)
RT112 (ARID1Akd)
VMCUB-1 (ARID1Amut)
HT1376 (ARID1Amut)
D
5637wt
5637-ARID1Akd
RT112wt
RT112-ARID1Akd
VMCUB-1(ARID1Amut)
HT1376(ARID1Amut)
CPI-1205
EPZ-6428
MAK-683
ARID1A
EZH2
H3K27Me3
Total H3
E
VMCUB-1 (ARID1Amut/empty vector)
Vehicle
GSK-126
VMCUB-1 (ARID1Aoe)
Vehicle
GSK-126

Supplementary Fig S5

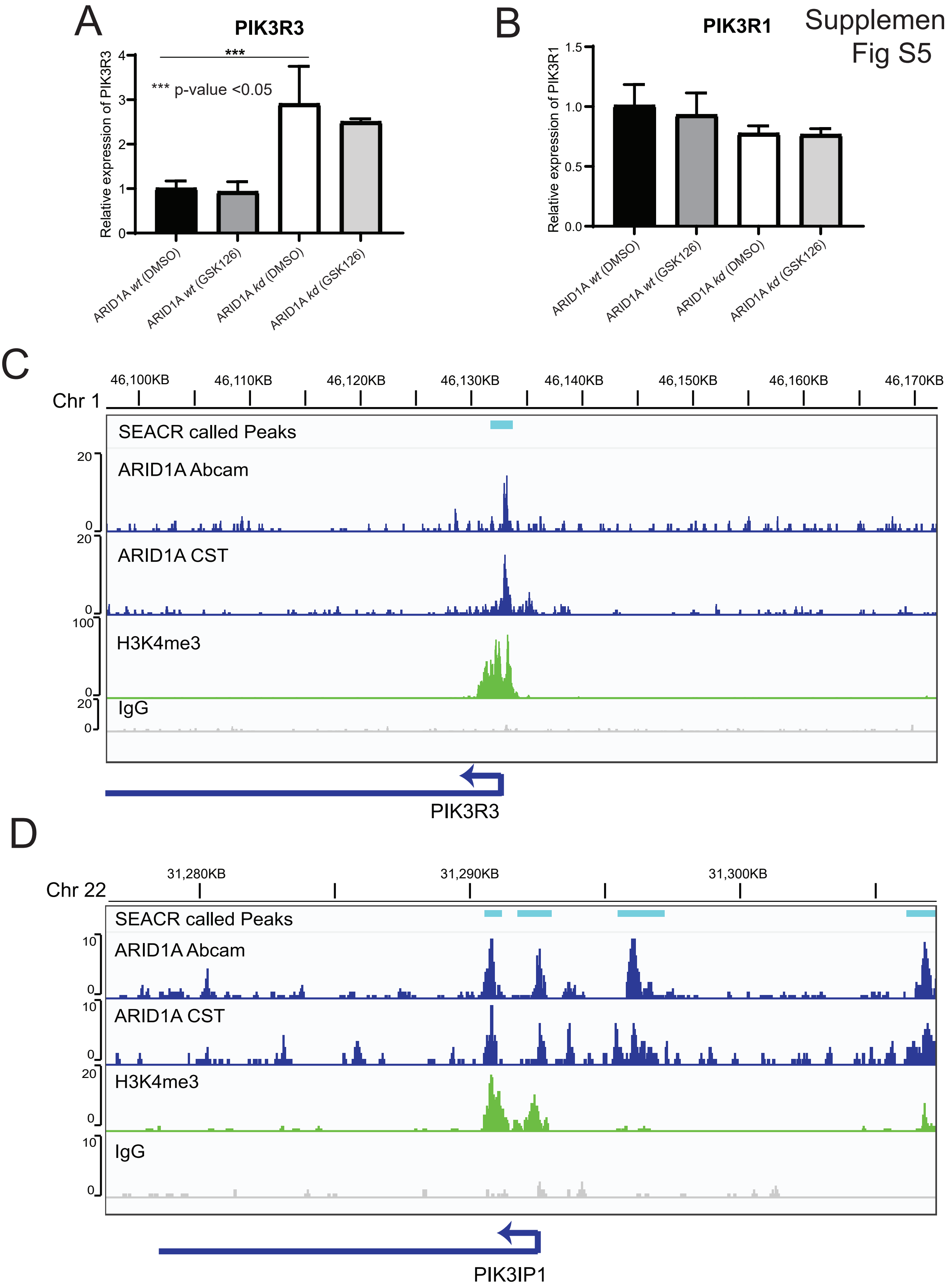

Supplementary Fig S6

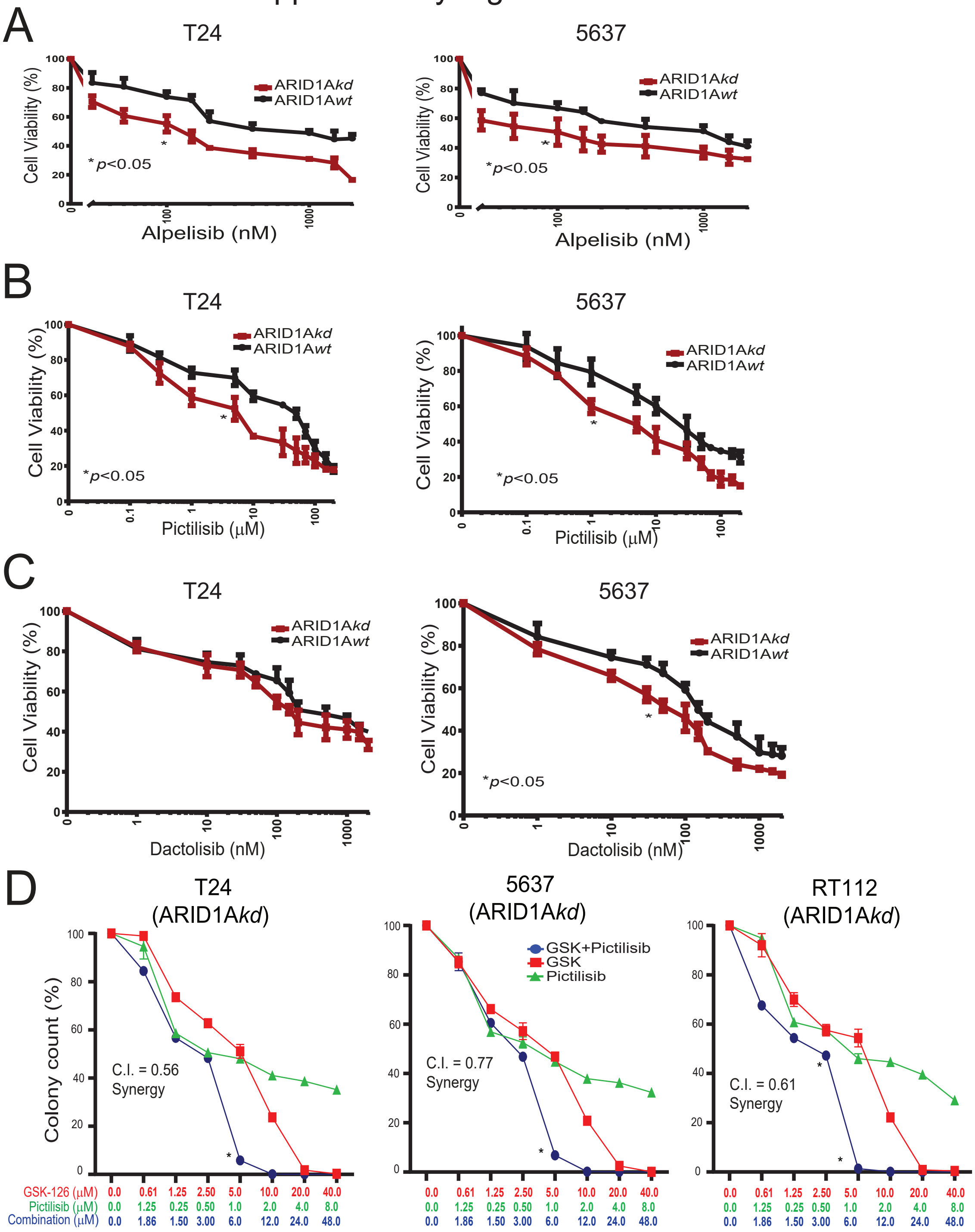

Supplementary Fig S7

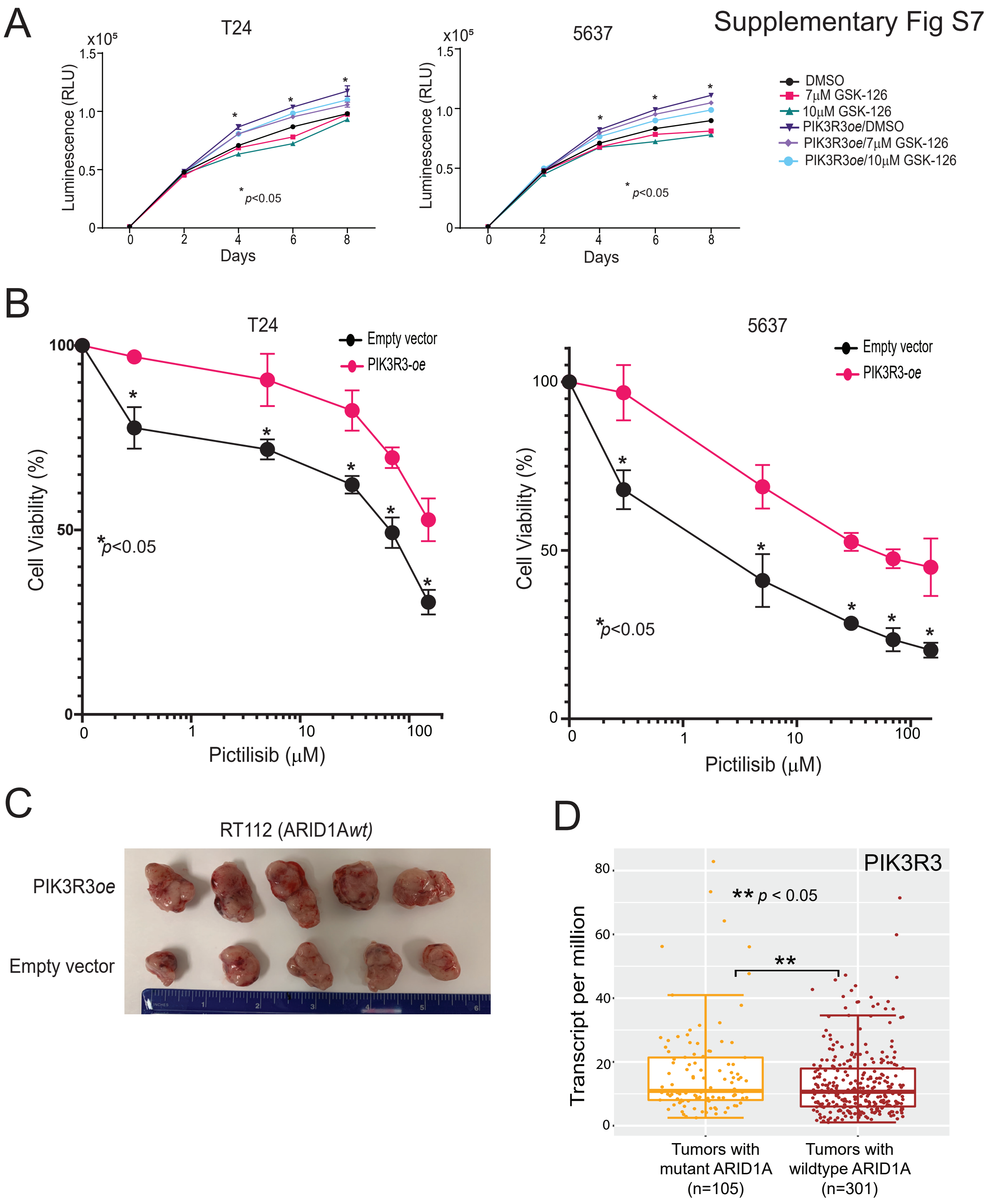

Supp Fig S8

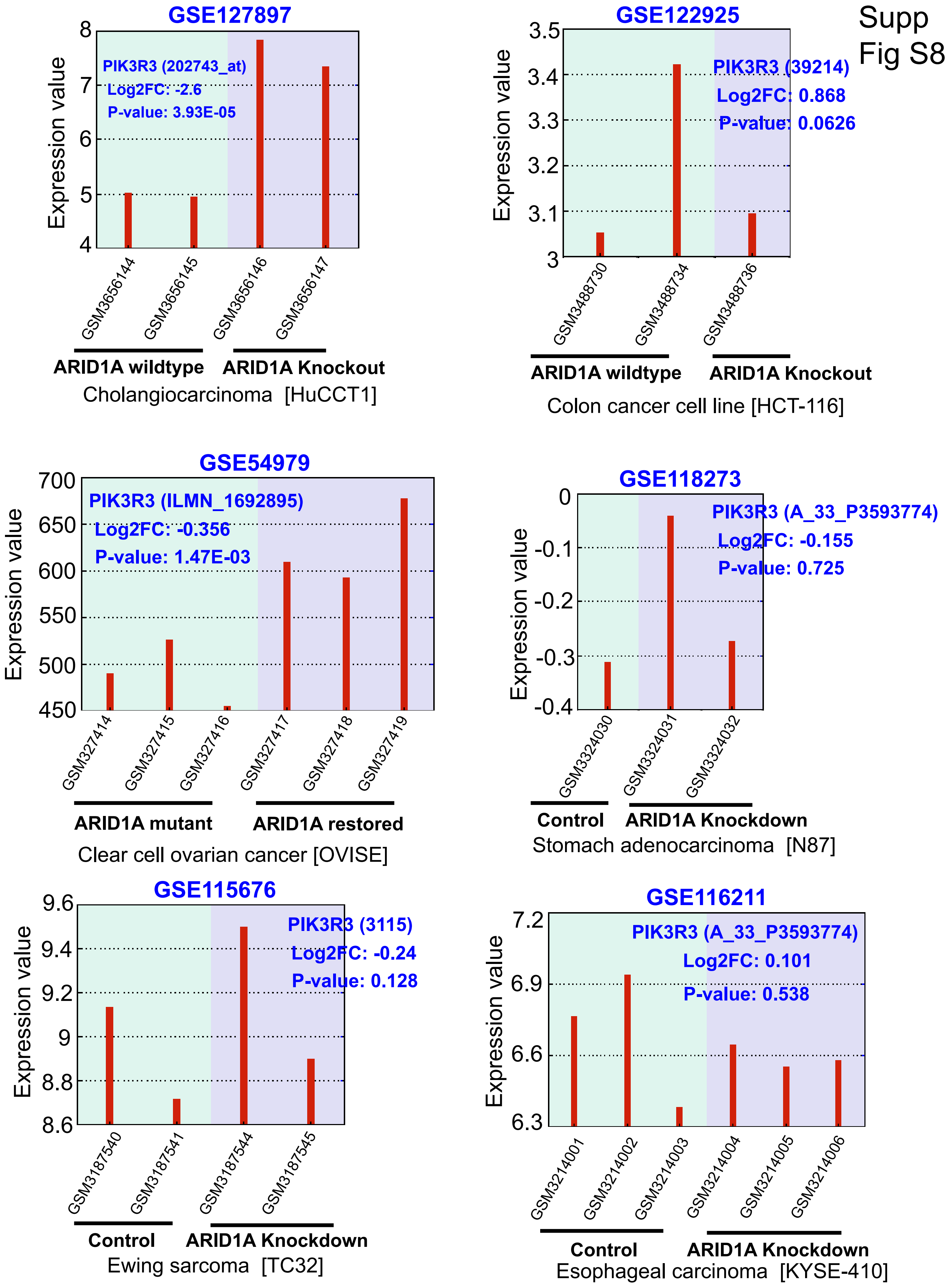
GSE127897
PIK3R3 (202743_at)
Log2FC: -2.6
P-value: 3.93E-05
Expression value
8
7
6
5
4
GSM3656144
GSM3656145
GSM3656146
GSM3656147
ARID1A wildtype
ARID1A Knockout
Cholangiocarcinoma [HuCCT1]
GSE122925
PIK3R3 (39214)
Log2FC: 0.868
P-value: 0.0626
Expression value
3.5
3.4
3.3
3.2
3.1
3
GSM3488730
GSM3488734
GSM3488736
ARID1A wildtype
ARID1A Knockout
Colon cancer cell line [HCT-116]
GSE54979
PIK3R3 (ILMN_1692895)
Log2FC: -0.356
P-value: 1.47E-03
Expression value
700
650
600
550
500
450
GSM327414
GSM327415
GSM327416
GSM327417
GSM327418
GSM327419
ARID1A mutant
ARID1A restored
Clear cell ovarian cancer [OVISE]
GSE118273
PIK3R3 (A_33_P3593774)
Log2FC: -0.155
P-value: 0.725
Expression value
0
-0.1
-0.2
-0.3
-0.4
GSM3324030
GSM3324031
GSM3324032
Control
ARID1A Knockdown
Stomach adenocarcinoma [N87]
GSE115676
PIK3R3 (3115)
Log2FC: -0.24
P-value: 0.128
Expression value
9.6
9.4
9.2
9
8.8
8.6
GSM3187540
GSM3187541
GSM3187544
GSM3187545
Control
ARID1A Knockdown
Ewing sarcoma [TC32]
GSE116211
PIK3R3 (A_33_P3593774)
Log2FC: 0.101
P-value: 0.538
Expression value
7.2
6.9
6.6
6.3
GSM3214001
GSM3214002
GSM3214003
GSM3214004
GSM3214005
GSM3214006
Control
ARID1A Knockdown
Esophageal carcinoma [KYSE-410]

Supplementary Figure Legends

Supplementary Figure S1: Bladder cancer harbors high rates of *ARID1A* mutation, and high expression of EZH2. (A) Oncoplots showing prevalence of ARID1A and EZH2 mutations in muscle invasive and non-muscle invasive bladder carcinomas. Mutation profiles from three independent genomic sequencing data gathered from cBioPortal.org show a significant number of bladder tumors harbor truncating or missense mutations in ARID1A. (B) Box-whisker plot showing expression level of EZH2 in TCGA bladder cancers with wild-type and mutant ARID1A alleles. T-test was performed.

Supplementary Figure S2: ARID1A mutation is associated with increased GSK-126 sensitivity. (A) Immunoblot analysis indicating that GSK-126 inhibits H3K27me3 generation in all cell lines (from experiment in Figure 1C). (B) Cell viability time course with increasing concentrations of GSK-126 in T24 (ARID1A*wt*) cell line. (C) Colony formation assay of ARID1A*mut* or wild type bladder cancer cell lines in the presence and absence of GSK-126 (6d). Photographic images and bar plots are shown. UT = untreated. T-tests were performed.

Supplementary Figure S3: GSK-126 treatment effectively inhibits H3K27 trimethylation in xenograft tissues, and inhibits xenograft growth in ARID1A*mut* cell lines. (A) Lysates from xenografts depicted in Figure 1D (n=5 per group) were used to perform immunoblots for H3K27me3, total H3 and β-actin in RT-112 (ARID1A*wt*) and HT1376 (ARID1A*mut*) cells. (B) Photographs of xenograft tumors (from Figure 1D) from mice inoculated with RT-112, 5637 (ARID1A*wt*), or HT1376 or VMCUB-1 (ARID1A*mut*) cells treated with and without GSK-126.

Supplementary Figure S4: ARID1A deficiency is necessary and sufficient for sensitivity to GSK-126. (A) Colony formation assay of 5637 ARID1A*kd* or ARID1A*wt* cells with or without GSK-126 treatment. (B) Photographs and immunoblots of xenograft tumors from mice (from Fig 2E) inoculated with RT112 (ARID1A*kd*) or 5637 (ARID1A*kd*). (C) Dose response cell viability experiments with various EZH2 inhibitors. Two-way ANOVA was performed comparing IC50 values of ARID1A*wt* and ARID1A*def* cells. (D) Immunoblots corresponding to cells in part (C) indicating effective EZH2 inhibition (via H3K27Me3) or degradation. (E) Photographs of endpoint xenograft tumors (from Fig 2H) from mice inoculated with VMCUB-1 (ARID1A*mut/*Empty vector) and VMCUB-1 (ARID1A reconstituted/overexpression) cells with and without GSK-126 treatment.

Supplementary Figure S5: ARID1A presence and effects at PIK3R3 and PIK3IP1 loci (A) Quantitative RT-PCR was performed to determine relative expression of PIK3R3 and (B) PIK3R1 in ARID1A*wt* and ARID1A*kd* RT112 cells in absence and presence of GSK-126 (5μM) after 24h treatment. No difference was seen after GSK-126 treatment. T-test was performed. (C) CUT & RUN analysis of PIK3R3 genomic locus using two different ARID1A antibodies (Abcam and Cell Signaling Technologies (CST)), H3K4me3 as an active mark, and IgG negative control. SEACR called peaks confirmed that ARID1A is present at PIK3R3 promoter. (D) CUT & RUN analysis of PIK3IP1 genomic locus using antibodies as in (C). SEACR called peaks confirmed that ARID1A is present at the PIK3IP1 promoter.

Supplementary Figure S6: ARID1A deficiency sensitizes bladder cancer cells to PI3K inhibitors. (A-C) Cell viability dose-response analysis in 5637 and T24 cells with ARID1A*kd* or empty vector (ARID1A*wt*) using alpelisib (a PI3K alpha-selective inhibitor), pictilisib (a PI3K class I selective inhibitor), and dactolisib (a dual PI3K/mTOR inhibitor) (all 48h). T-tests were performed on IC50 values. (D) Colony formation dose-response assay using increasing doses of GSK-126, pictilisib, or combination showed that the combination was synergistic. Combination Indices (CI) were calculated by the Chou-Talalay method.

Supplementary Figure S7: PIK3R3 overexpression increases growth kinetics of ARID1A*wt* cells. (A) ARID1A*wt* cells T24 and 5637 with PIK3R3 overexpression (oe) show increased proliferation at baseline (compared with empty vector), but no increased sensitivity to GSK-126. Two-way ANOVA was performed. (B) Dose-response cell viability assay using T24 and 5637 ARID1A*wt* cells with PIK3R3 overexpression (oe) or empty vector showing that PIK3R3 overexpression causes resistance to the PI3K inhibitor, pictilisib (48 h). T-tests were performed. (C) Photographs of end-point xenograft tumors (from Fig 7G) from mice inoculated with RT112 (ARID1A*wt*) over-expressing PIK3R3 compared to empty vector. (D) Box-plot analysis of TCGA bladder cancer data comparing PIK3R3 mRNA levels between ARID1A*mut* and ARID1A*wt* tumors. T-test was performed.

Supplementary Figure S8: PIK3R3 expression pattern in ARID1A mutated/altered cancer cell lines. GEO2R profile graphs showing expression level of PIK3R3 in ARID1A altered ovarian cancer (OVISE), colon cancer (HCT-116), cholangiocarcinoma (HuCCT1), stomach adenocarcinoma (N87), Ewing sarcoma (TC32) and esophageal carcinoma (KYSE-410) cell lines. Expression value from each sample is shown in addition to probe identifiers, log 2 fold change and t-test p-value. A 2 fold change was considered significant.

Supplementary Methodology

In silico data analysis

Using cBioPortal, the mutation profiles of ARID1A and EZH2 in muscle-invasive and non-muscle invasive bladder cancer were obtained (1). cBioPortal provides user-friendly graphical interface to analyze whole exome sequencing datasets from The Cancer Genome Atlas Project and other published studies (2-4). To study EZH2 gene expression profile in invasive bladder carcinoma patients, TCGA level 3 RNA-seq data (including "raw_read_count" and "scaled_estimate" for each sample) was downloaded for all primary tumor and normal samples using TCGA-Assembler (5). Transcript per million values for each gene was obtained by multiplying scaled estimate by 1,000,000. Using patient ID from cBioPortal, primary tumors were categorized based on ARID1A mutation status. Boxplot was generated using R (https://cran.r-project.org/).

We studied the expression pattern of PIK3R3 in ARID1A altered/mutated cancer cell lines. Microarray datasets related to OVISE [GSE54979], HCT-116 [GSE122925], N87 [GSE118273], HuCCT1 [GSE127897], TC32 [GSE115676] and KYSE410 [GSE116211] cell lines were analyzed using GEO2R tool (https://www.ncbi.nlm.nih.gov/geo/geo2r/)(6-11). Differential expression analysis between ARID1A mutated/altered and control samples was performed for each study separately. Profile graphs, log2 fold change and p-value was obtained for probes related to PIK3R3.

Colony formation assay

Bladder cancer cells were seeded at 800 cells per well of 6-well plates (triplicate) and incubated at 37 °C with 5% CO_2 for 7–10 days while treating with GSK126 or pictilisib at the doses indicated every two days. Here both untreated and DMSO treated cells served as controls. Colonies were fixed with 10% (v/v) ethanol for 30 min and stained with crystal violet (Sigma-Aldrich, St Louis, MO, USA) for 20 min. Then, the photographs of the colonies were taken using Amersham Imager 600RGB (GE Healthcare Life Sciences, Pittsburgh, PA, USA). Colony quantification was carried out using ImageQuant TL Colony v.8.1 software (GE Healthcare Life Sciences).

qRT-PCR analysis

RNA from cultured cells was extracted with Direct-zol RNA miniprep kit (Zymo Research). For qRT-PCR, cDNA was generated using a High-Capacity cDNA Reverse Transcription kit (Applied Biosystems). qRT-PCR analysis was performed using the Taqman Gene Expression Master reagent mixed with Taqman primers and analyzed with the QuantStudioTM 6K Flex Real-Time PCR System (Applied Biosystems). mRNA expression levels were normalized to human TATA-binding protein (TBP) or GAPDH, and the normalized cycle threshold (Ct) values were quantified using the double delta Ct analysis. qRT-PCR data represent relative expression. In general, controls in experimental data are normalized to a value of 1. Indicated Taqman primers were predesigned from Applied Biosystems as follows: *PIK3R1* (Hs00933163_m1), *PIK3R3* (Hs01103591_m1), *GAPDH* (Hs02786624_g1), *TBP* (Hs00427620_m1).

Protein extraction and western blotting of Xenograft tissues

The mice were euthanized, and the xenograft tumors were excised and weighed. Next, fragments of xenograft tumors were lysed in RIPA lysis buffer (Thermo Scientific, IL, USA) and boiled for 10 min at 90 °C. Protein concentrations were measured using the BCA assay. Approximately, 50 μg of protein extract from fresh surgical xenograft tissues were separated by 10% SDS-polyacrylamide gel electrophoresis (SDS-PAGE). The gels were then electrotransferred onto polyvinylidene difluoride (PVDF) membranes (EMD Millipore, Billerica, MA, USA).

CUT&RUN library preparation

RT112 cells were harvested using Accutase (Gibco) and slow frozen in aliquots containing 500,000 cells in serum-containing DMEM+10% DMSO. For CUT&RUN, cell aliquots were rapidly thawed at 37°C and washed as directed. Samples were processed in a single batch using the CUTANA CUT&RUN Kit (Epicypher) according to the manufacturer's instructions and using 1 ng e. coli spike-in DNA per sample. After DNA purification, DNA libraries were prepared using the NEBNext Ultra II DNA Library Prep Kit for Illumina (NEB) and NEBNext Multiplex Oligos for Illumina (96 Unique Dual Index Primer Pairs, NEB) with samples randomized into two library preparation batches. Library quality was assessed using Qubit (Thermo Fisher) and 4150 TapeStation System (Agilent). All libraries were pooled and sequenced on a NextSeq 550 (Illumina) using a NextSeq 500/550 v2.5 Mid Output Kit (150 cycles, paired-end, Illumina).

CUT&RUN data processing and analysis

Sequencing reads were demultiplexed and converted to FASTQ format using bcl2fastq2 (Illumina). Adapter sequences were trimmed (cutadapt) and reads were aligned to both the human (GRCh38) and E. Coli K-12 MG1655 genomes (bwa-mem2). PCR duplicates were removed (Picard) and non-uniquely mapping reads were filtered out (samtools). Replicates were scaled to each other using the ratio of E. coli reads to total reads and scaled BigWig files were generated for data visualization (bedtools bamcoverage). Alignment files were converted to paired-end BED files and then full-fragment BED files (bedtools bamtobed, awk, cut) and scaled BedGraph files were generated using the same scaling strategy as for the BigWig files (deeptools genomecoverage). Peaks were called from the scaled BedGraph files using SEACR (12) in relaxed mode to facilitate broad discovery of potential peaks; the top 0.001% of peaks were retained for analysis. Replicate peaks were merged (bedtools merge), annotated (ChIPpeakAnno) for the closest TSS, and visualized in IGV. The CUT&RUN data generated in this study are publicly available in Gene Expression Omnibus (GEO) at GSE203033.

Table 1

Antibody	Dilution	Supplier	Cat. No.	RRID
ARID1A	1:1000 (IB), 1:10 (CUT&RUN, CNR)	Cell Signaling Technologies, Denver, MA	12354S	AB_2637010

ARID1A	1:100 (CNR)	Abcam, Waltham, MA	ab182560	AB_2889973
BRG1	1:1000	Cell Signaling Technologies, Denver, MA	49360S	AB_2728743
BRM	1:1000	Cell Signaling Technologies, Denver, MA	11966S	AB_2797783
BAF47	1:1000	Cell Signaling Technologies, Denver, MA	91735S	AB_2800172
EZH2	1:1000	Cell Signaling Technologies, Denver, MA	5246S	AB_10694683
IgG	1:50 (CNR)	EpiCypher, Durham, NC	13-0042	
H3K4me3	1:50 (CNR)	EpiCypher, Durham, NC	13-0041	
H3K27me3	1:2000	Cell Signaling Technologies, Denver, MA	9733S	AB_2616029
p-AKT (T308)	1:1000	Cell Signaling Technologies, Denver, MA	13038	AB_2629447
p-AKT (S473)	1:1000	Cell Signaling Technologies, Denver, MA	9271	AB_329825
AKT	1:1000	Cell Signaling Technologies, Denver, MA	9272	AB_329827
Phospho-mTOR	1:1000	Cell Signaling Technologies, Denver, MA	2971	AB_330970
Phospho-p70 S6 Kinase	1:1000	Cell Signaling Technologies, Denver, MA	9205	AB_330944
p70 S6 Kinase	1:1000	Cell Signaling Technologies, Denver, MA	9202	AB_331676
Phospho-4E-BP1 (Thr37/46)	1:1000	Cell Signaling Technologies, Denver, MA	2855	AB_560835
4E-BP1	1:1000	Cell Signaling Technologies, Denver, MA	9452	AB_331692
PI3 Kinase p110α	1:1000	Cell Signaling Technologies, Denver, MA	4255	AB_659888
PI 3-kinase p110β	1:1000	Santa Cruz Biotechnology, CA	376641	AB_11150840
p38 MAPK	1:1000	Cell Signaling Technologies, Denver, MA	9212	AB_330713
Phospho-p38 MAPK (Thr180/Tyr182)	1:1000	Cell Signaling Technologies, Denver, MA	4631	AB_331765
p44/42 MAPK (Erk1/2)	1:1000	Cell Signaling Technologies, Denver, MA	4695	AB_390779
Phospho-p44/42 MAPK (Erk1/2) (Thr202/Tyr204)	1:1000	Cell Signaling Technologies, Denver, MA	4376	AB_331772
Phospho-SAPK/JNK (Thr183/Tyr185)	1:1000	Cell Signaling Technologies, Denver, MA	4668	AB_823588
SAPK/JNK	1:1000	Cell Signaling Technologies, Denver, MA	9252	AB_2250373
Cleaved Caspase 3	1:1000	Cell Signaling Technologies, Denver, MA	9661	AB_2341188
LC3B (D11) XP	1:1000	Cell Signaling Technologies, Denver, MA	3868	AB_2137707
PTEN	1:1000	Cell Signaling Technologies, Denver, MA	9559	AB_390810
PIK3IP1	1:500	Proteintech Group, Inc, Rosemont, IL	16826-1-AP	AB_2163333

PIK3R1 (p85α/p55α/p50α)	1:1000	Abcam, Waltham, MA	ab191606	AB_2891324
PIK3R2 (p85β)	1:1000	Abcam, Waltham, MA	ab28356	AB_777259
PIK3R3 (p55γ)	1:1000	Santa Cruz Biotechnology	sc-376615	AB_11150683
Histone H3	1:2000	Cell Signaling Technologies, Denver, MA	4499	AB_10544537
Ubiquitin	1:1000	Life Sensors	VU-101	AB_2716558
β-actin	1:20000	Proteintech Group, Inc, Rosemont, IL	60008	AB_2883409
GAPDH	1:20000	Proteintech Group, Inc, Rosemont, IL	60004	AB_2107436
Anti-rabbit	1:5000	Proteintech Group, Inc, Rosemont, IL	SA00001-2	AB_2722564
Anti-mouse	1:5000	Proteintech Group, Inc, Rosemont, IL	SA00001-1	AB_2722565

Table 2:

S.No.	SiRNA Information	Lot. Number
1.	ON-TARGETplus Human ARID1A siRNA (Gene id:8289)	L-017263-00-0005
2.	ON-TARGETplus Human SMARCB1 siRNA (Gene id:6598)	L-010536-00-0005
3.	ON-TARGETplus Human SMARCA2 siRNA (Gene id:6595)	L-017253-00-0005
4.	ON-TARGETplus Human SMARCA4 siRNA (Gene id:6597)	L-010431-00-0005

References

1. Gao J, Aksoy BA, Dogrusoz U, Dresdner G, Gross B, Sumer SO, et al. Integrative analysis of complex cancer genomics and clinical profiles using the cBioPortal. Sci Signal. 2013;6(269):pl1.
2. Kim PH, Cha EK, Sfakianos JP, Iyer G, Zabor EC, Scott SN, et al. Genomic predictors of survival in patients with high-grade urothelial carcinoma of the bladder. Eur Urol. 2015;67(2):198-201.
3. Pietzak EJ, Bagrodia A, Cha EK, Drill EN, Iyer G, Isharwal S, et al. Next-generation Sequencing of Nonmuscle Invasive Bladder Cancer Reveals Potential Biomarkers and Rational Therapeutic Targets. Eur Urol. 2017;72(6):952-9.
4. Robertson AG, Kim J, Al-Ahmadie H, Bellmunt J, Guo G, Cherniack AD, et al. Comprehensive Molecular Characterization of Muscle-Invasive Bladder Cancer. Cell. 2017;171(3):540-56 e25.
5. Wei L, Jin Z, Yang S, Xu Y, Zhu Y, Ji Y. TCGA-assembler 2: software pipeline for retrieval and processing of TCGA/CPTAC data. Bioinformatics. 2018;34(9):1615-7.
6. Bitler BG, Aird KM, Garipov A, Li H, Amatangelo M, Kossenkov AV, et al. Synthetic lethality by targeting EZH2 methyltransferase activity in ARID1A-mutated cancers. Nat Med. 2015;21(3):231-8.
7. Ogiwara H, Takahashi K, Sasaki M, Kuroda T, Yoshida H, Watanabe R, et al. Targeting the Vulnerability of Glutathione Metabolism in ARID1A-Deficient Cancers. Cancer Cell. 2019;35(2):177-90 e8.
8. Ashizawa M, Saito M, Min AKT, Ujiie D, Saito K, Sato T, et al. Prognostic role of ARID1A negative expression in gastric cancer. Sci Rep. 2019;9(1):6769.
9. Selvanathan SP, Graham GT, Grego AR, Baker TM, Hogg JR, Simpson M, et al. EWS-FLI1 modulated alternative splicing of ARID1A reveals novel oncogenic function through the BAF complex. Nucleic Acids Res. 2019;47(18):9619-36.

10. Yoshino J, Akiyama Y, Shimada S, Ogura T, Ogawa K, Ono H, et al. Loss of ARID1A induces a stemness gene ALDH1A1 expression with histone acetylation in the malignant subtype of cholangiocarcinoma. Carcinogenesis. 2020;41(6):734-42.
11. Luo Q, Wu X, Chang W, Zhao P, Zhu X, Chen H, et al. ARID1A Hypermethylation Disrupts Transcriptional Homeostasis to Promote Squamous Cell Carcinoma Progression. Cancer Res. 2020;80(3):406-17.
12. Meers MP, Tenenbaum D, Henikoff S. Peak calling by Sparse Enrichment Analysis for CUT&RUN chromatin profiling. Epigenetics & chromatin. 2019;12(1):42.

Uncut blot Figure 1A

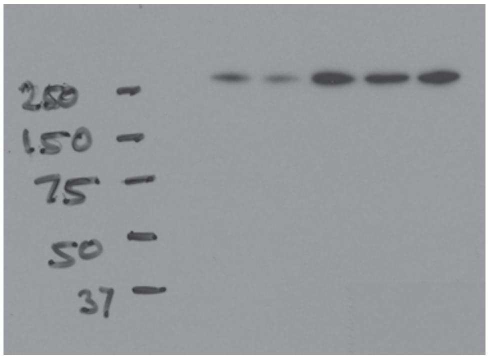

ARID1A

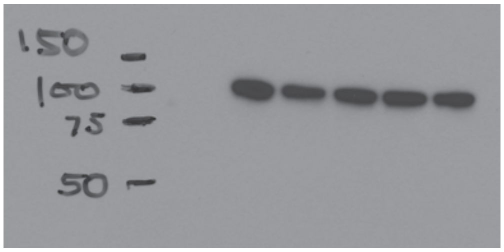

EZH2

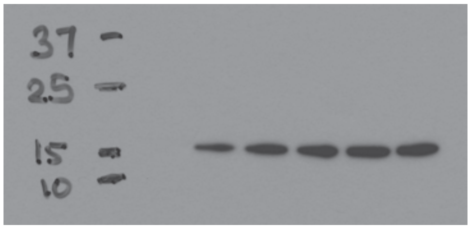

H3K27Me3

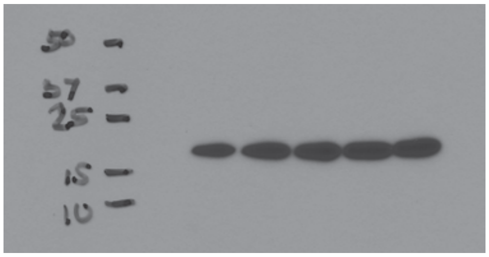

Total H3

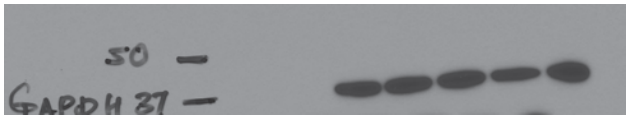

GAPDH

Uncut blot Figure 2A

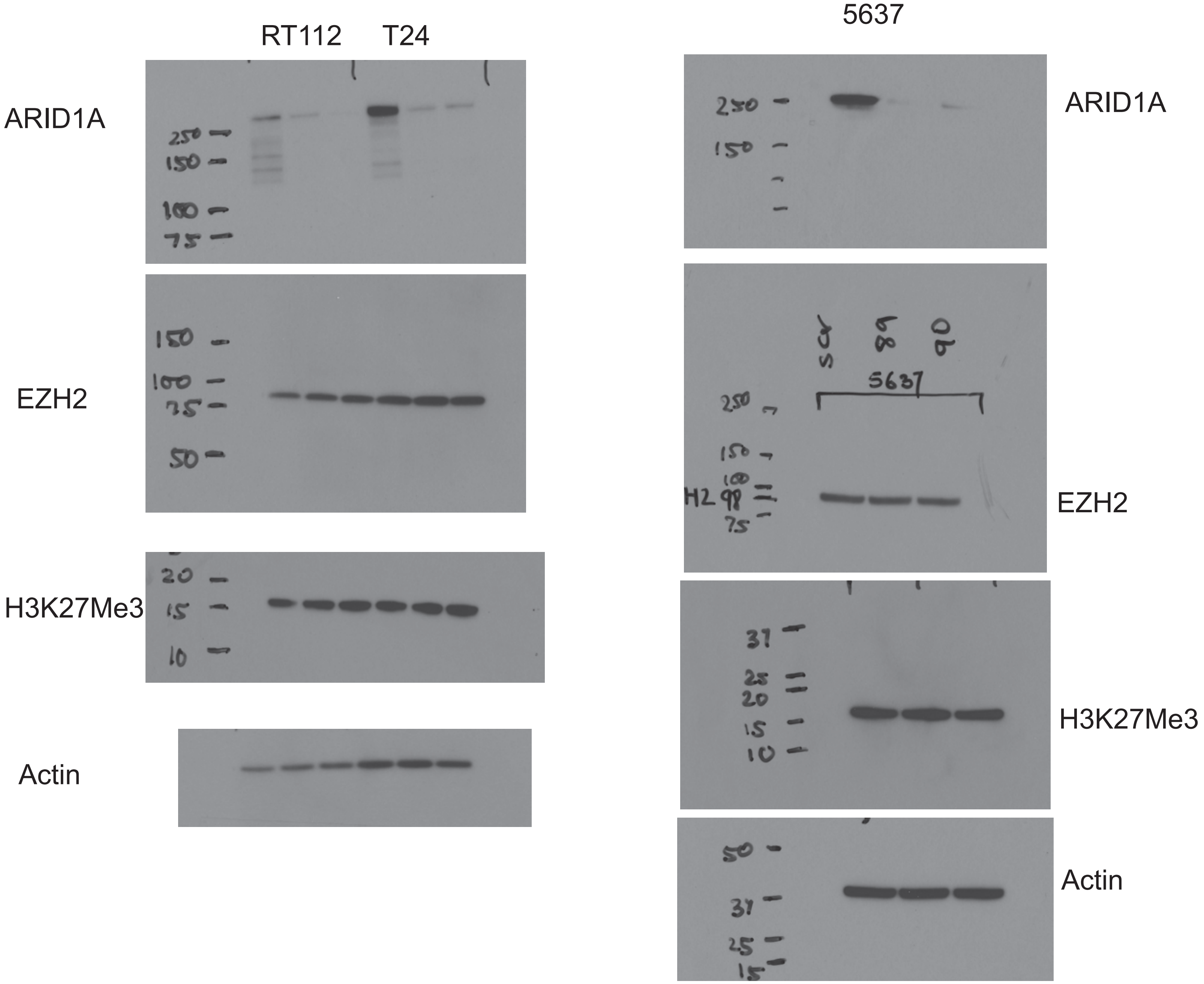

Uncut blots (Figure 2D)

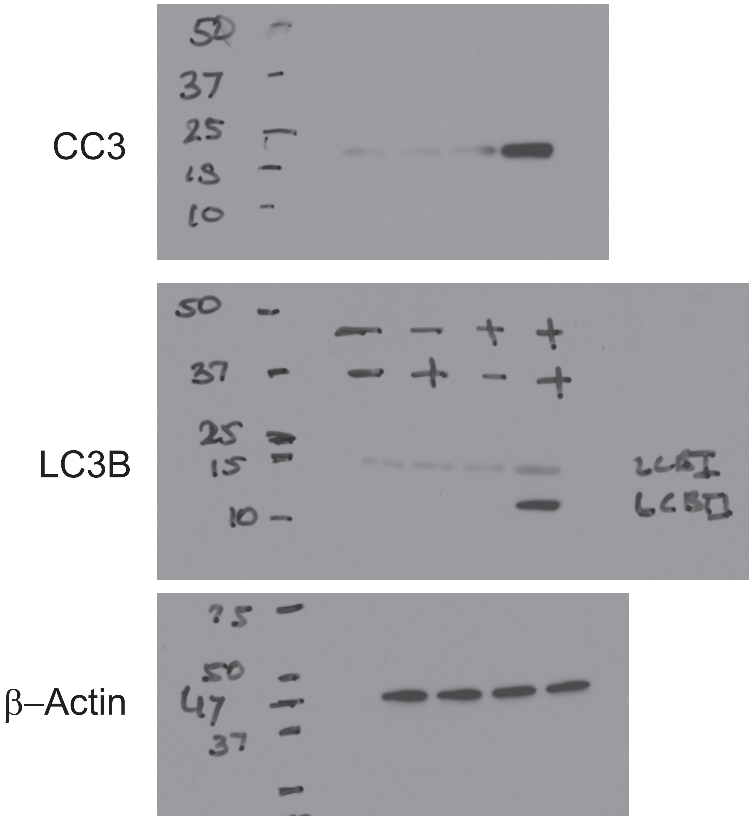

Uncut blot Figure 2F

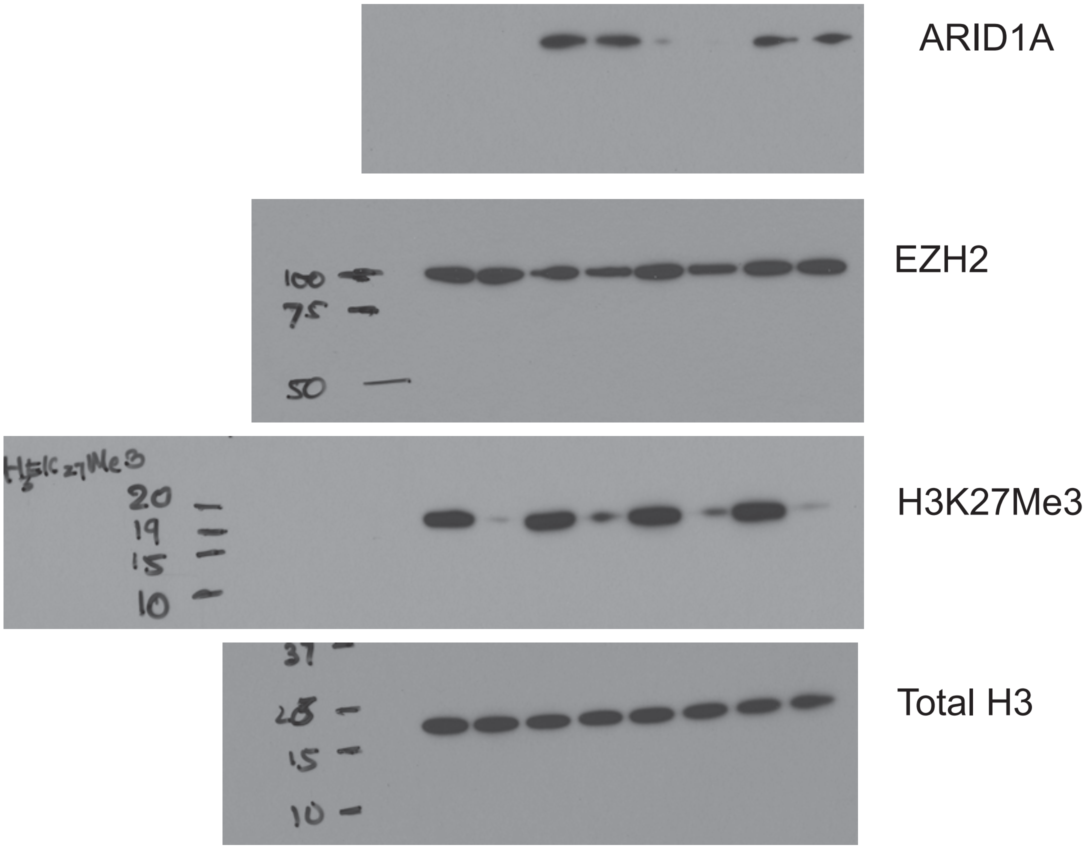

Uncut blot Figure 3C

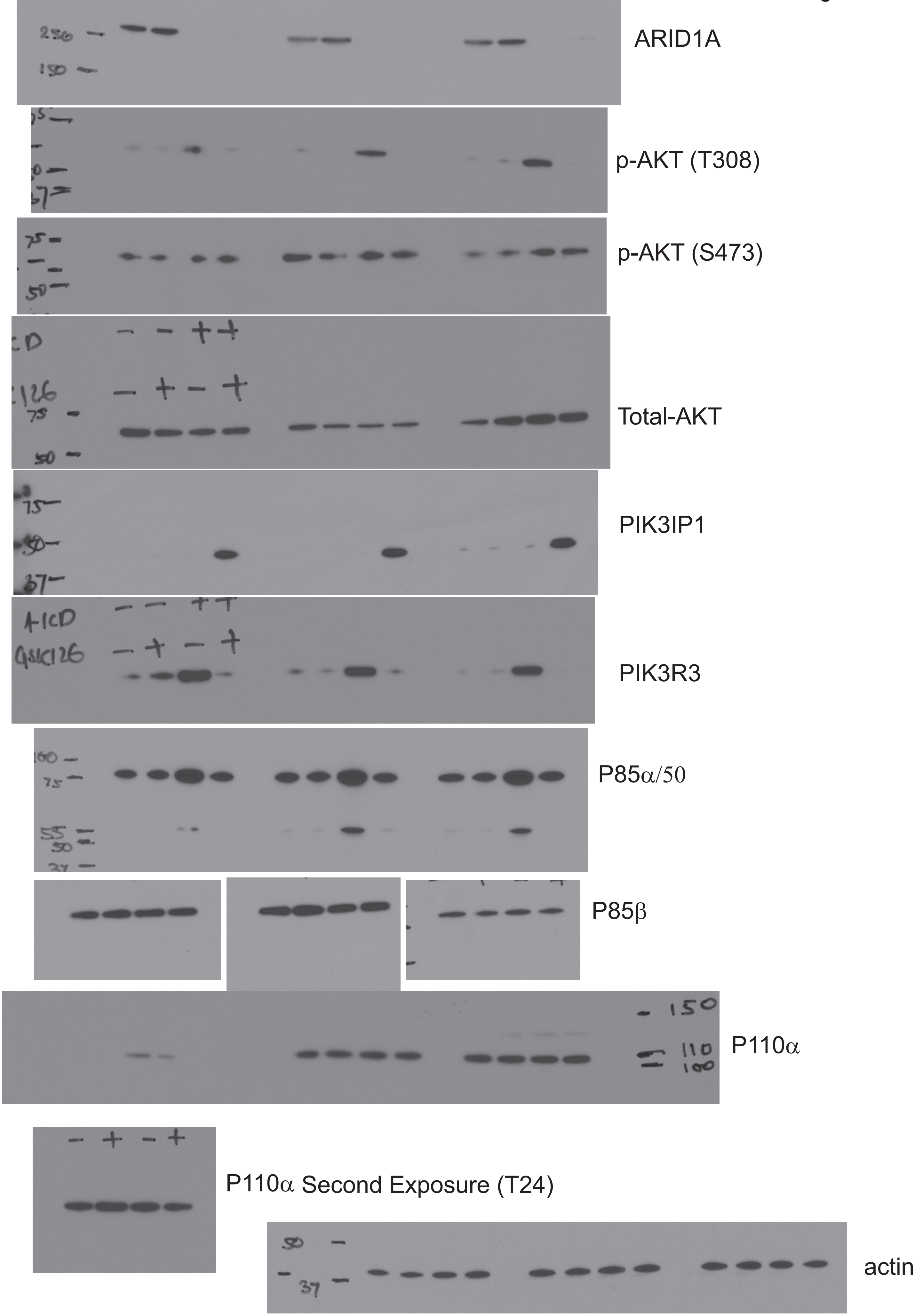

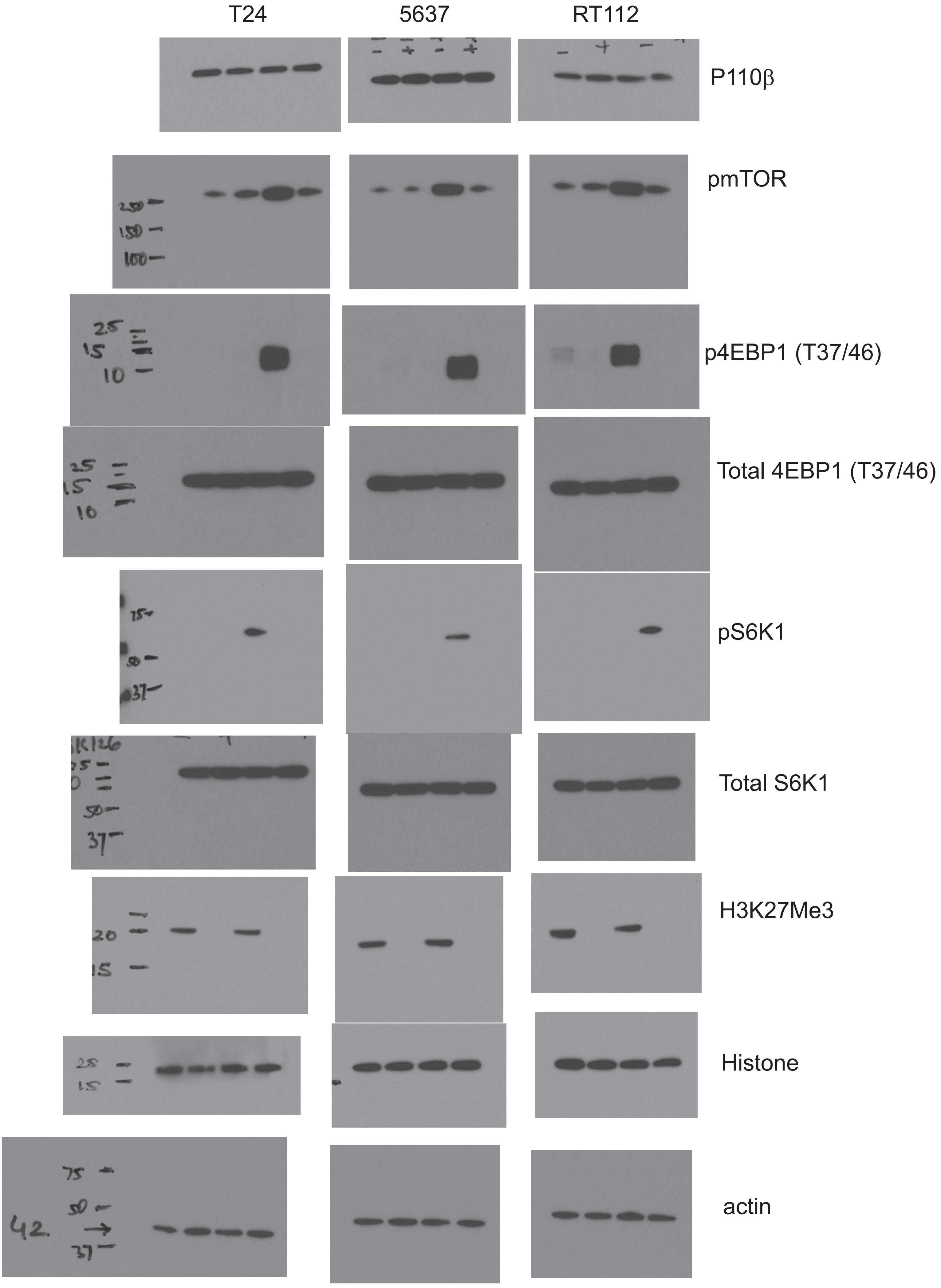

T24
5637
RT112
P110β
pmTOR
p4EBP1 (T37/46)
Total 4EBP1 (T37/46)
pS6K1
Total S6K1
H3K27Me3
Histone
actin

Uncut blot Figure 3D

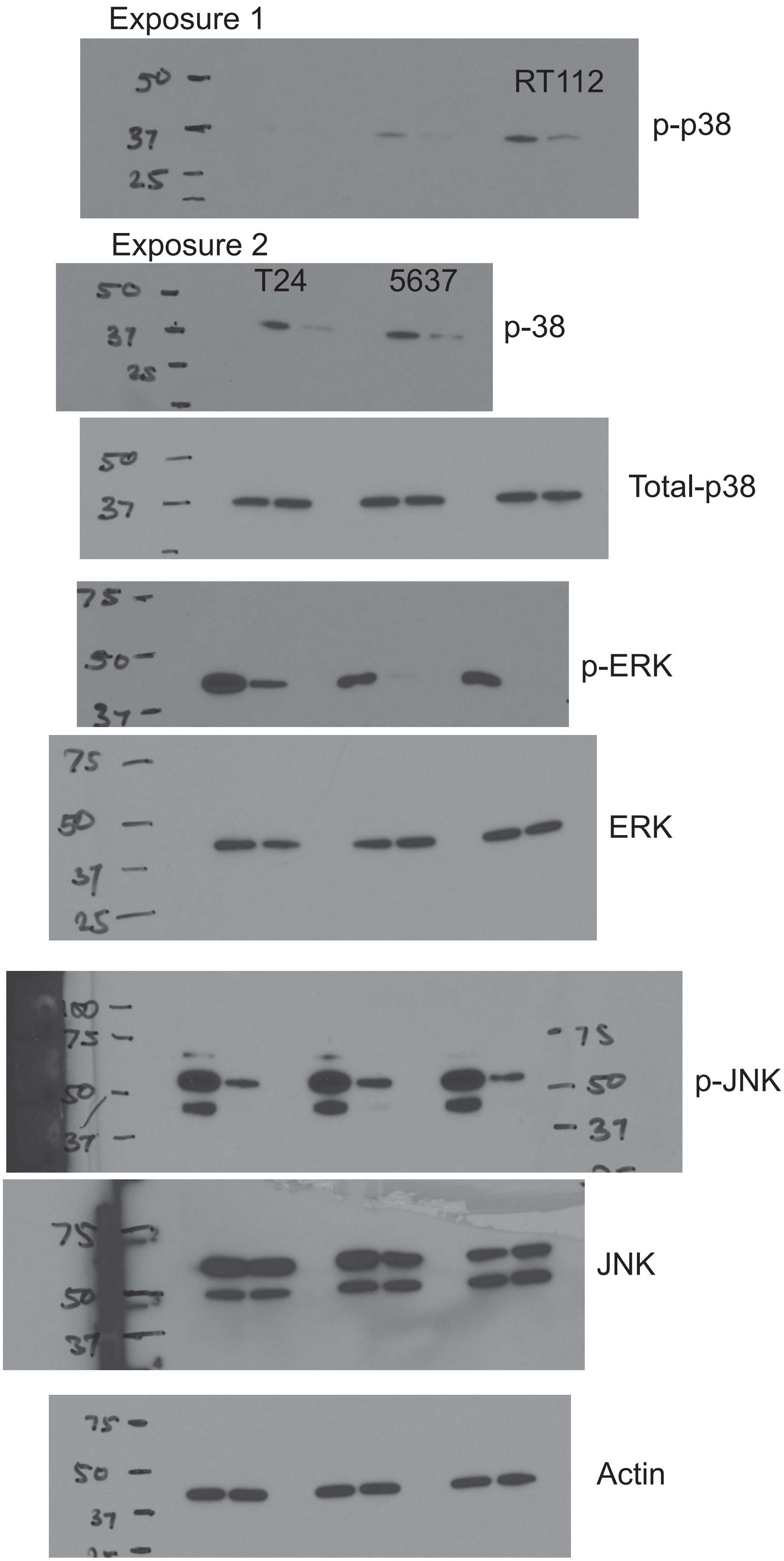

Uncut blot Figure 3E

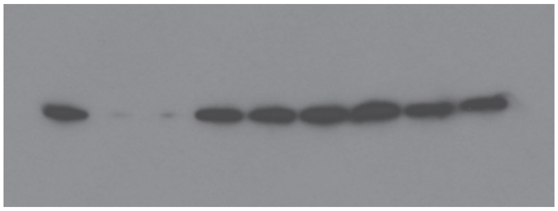

ARID1A_siRNA

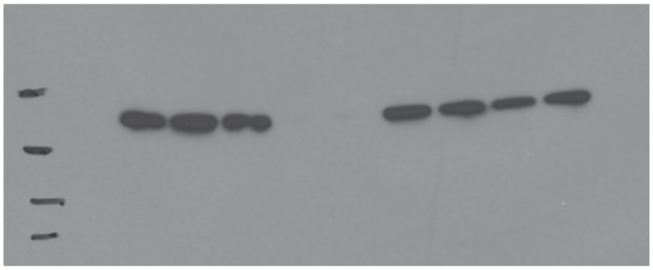

BRG1_siRNA

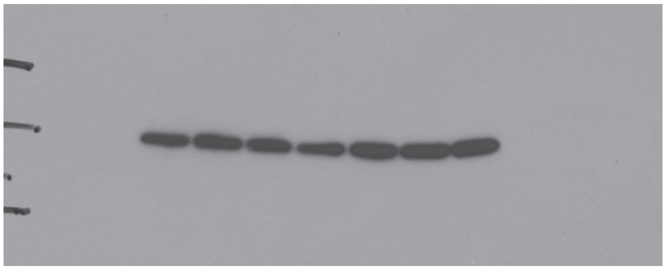

BRM_siRNA

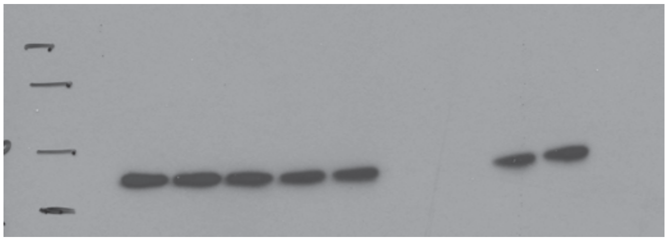

BAF47_siRNA

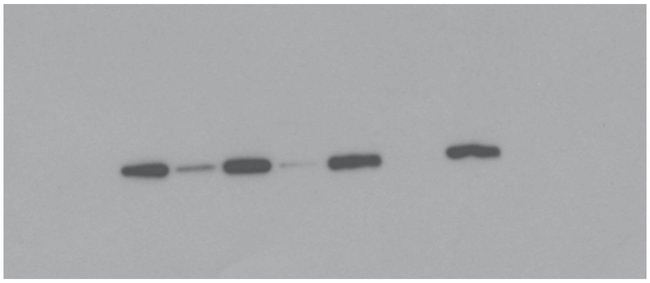

PIK3R3

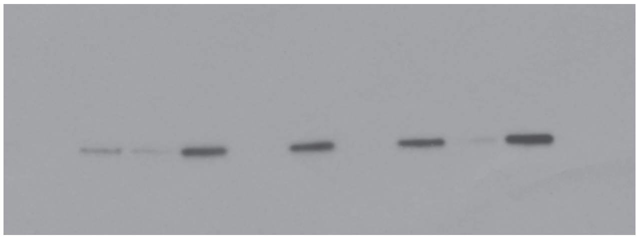

PIK3IP1

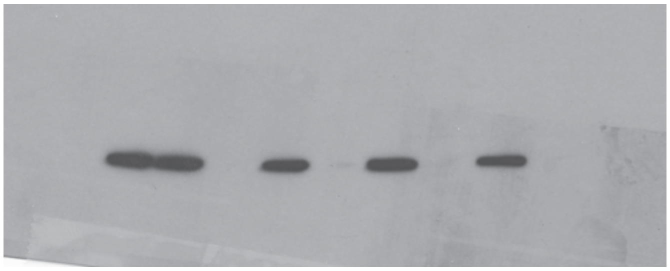

H3k27Me3

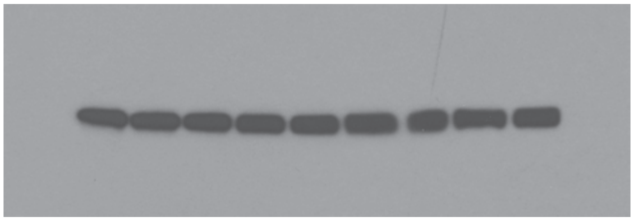

actin

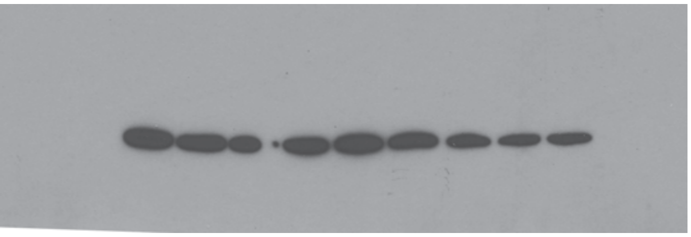

Total Histone

Uncut blot Figure 4A

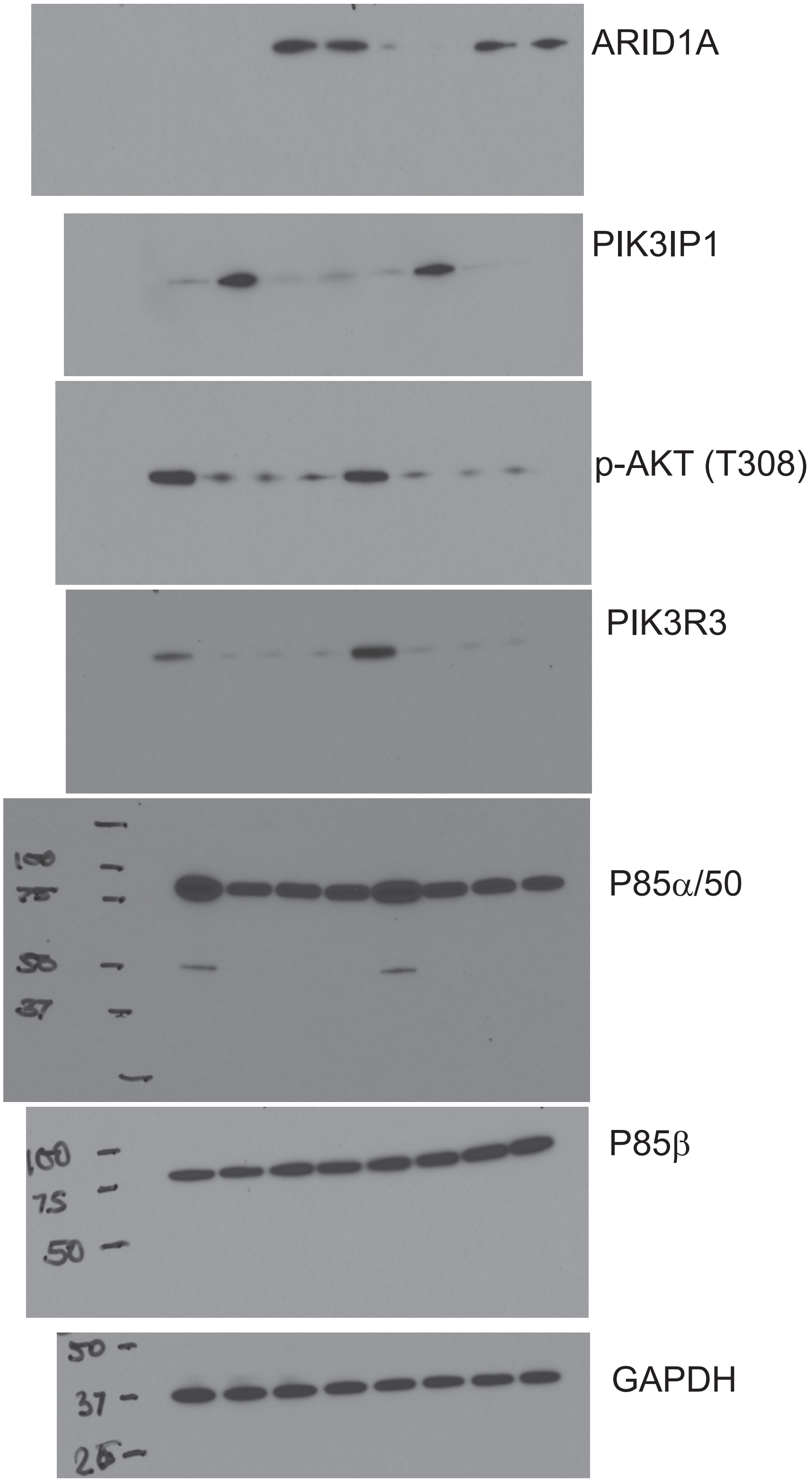

Uncut blot Figure 4B

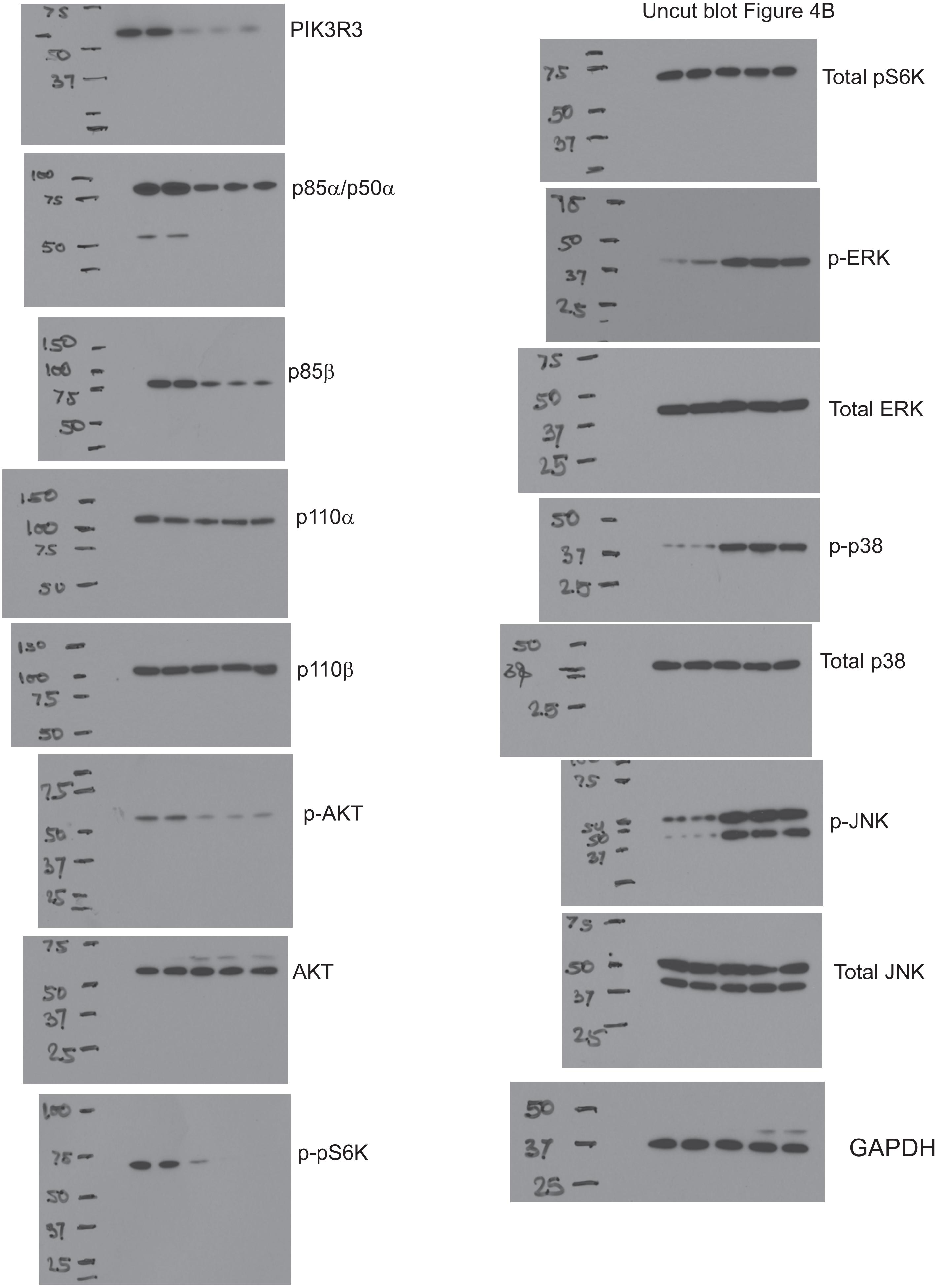

Uncut blot Figure 4C

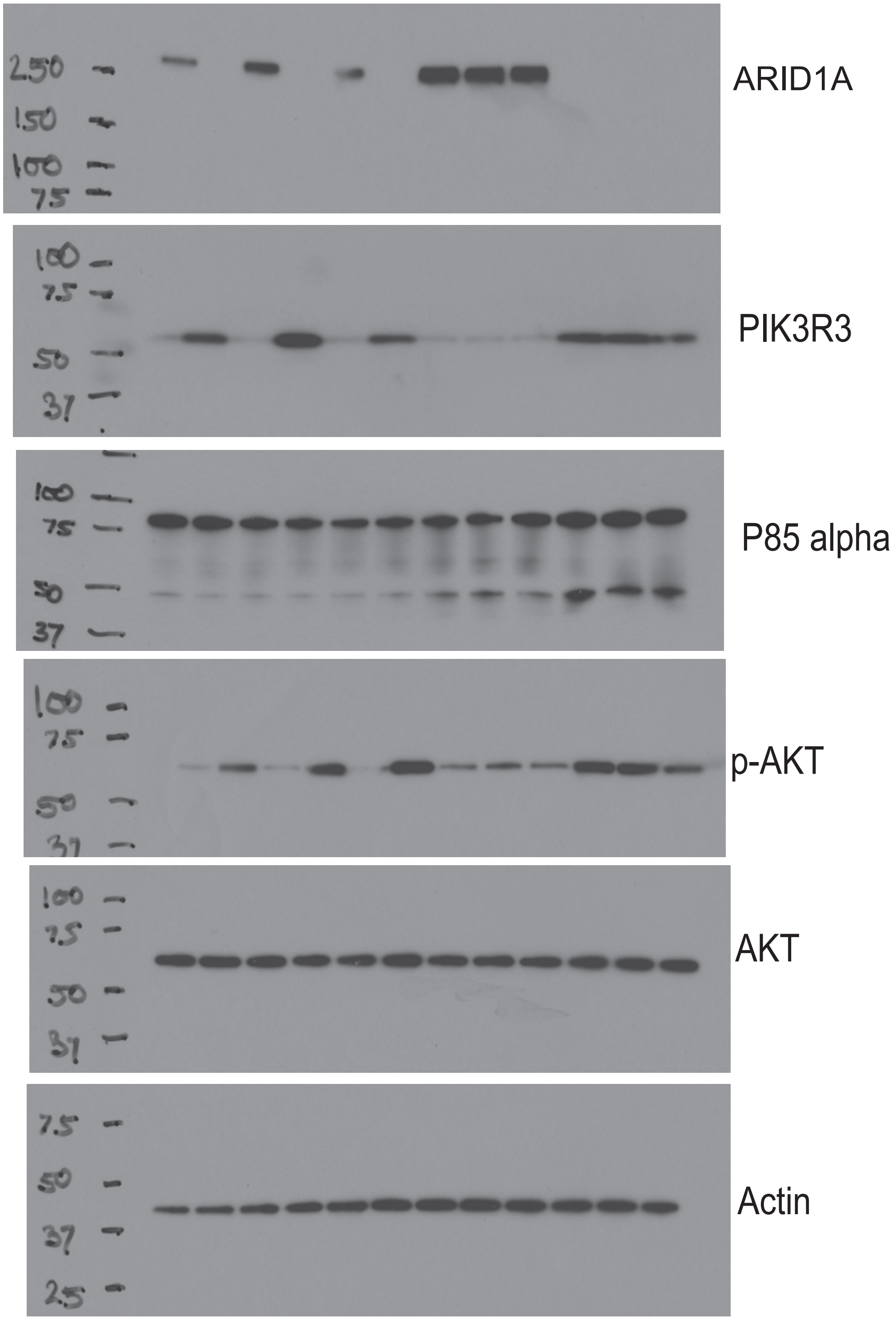

Uncut blot Figure 4D

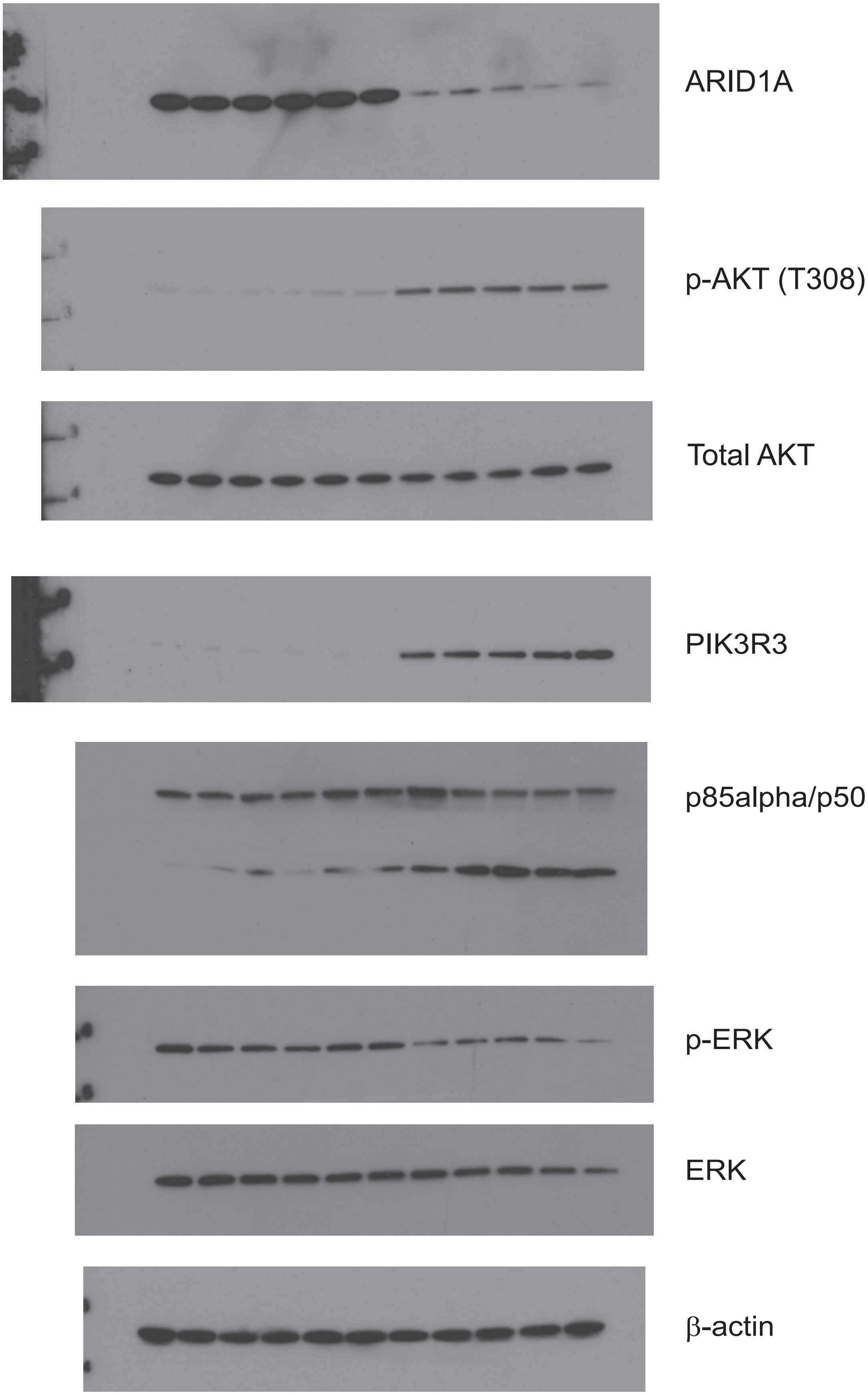

Uncut blot Figure 5A

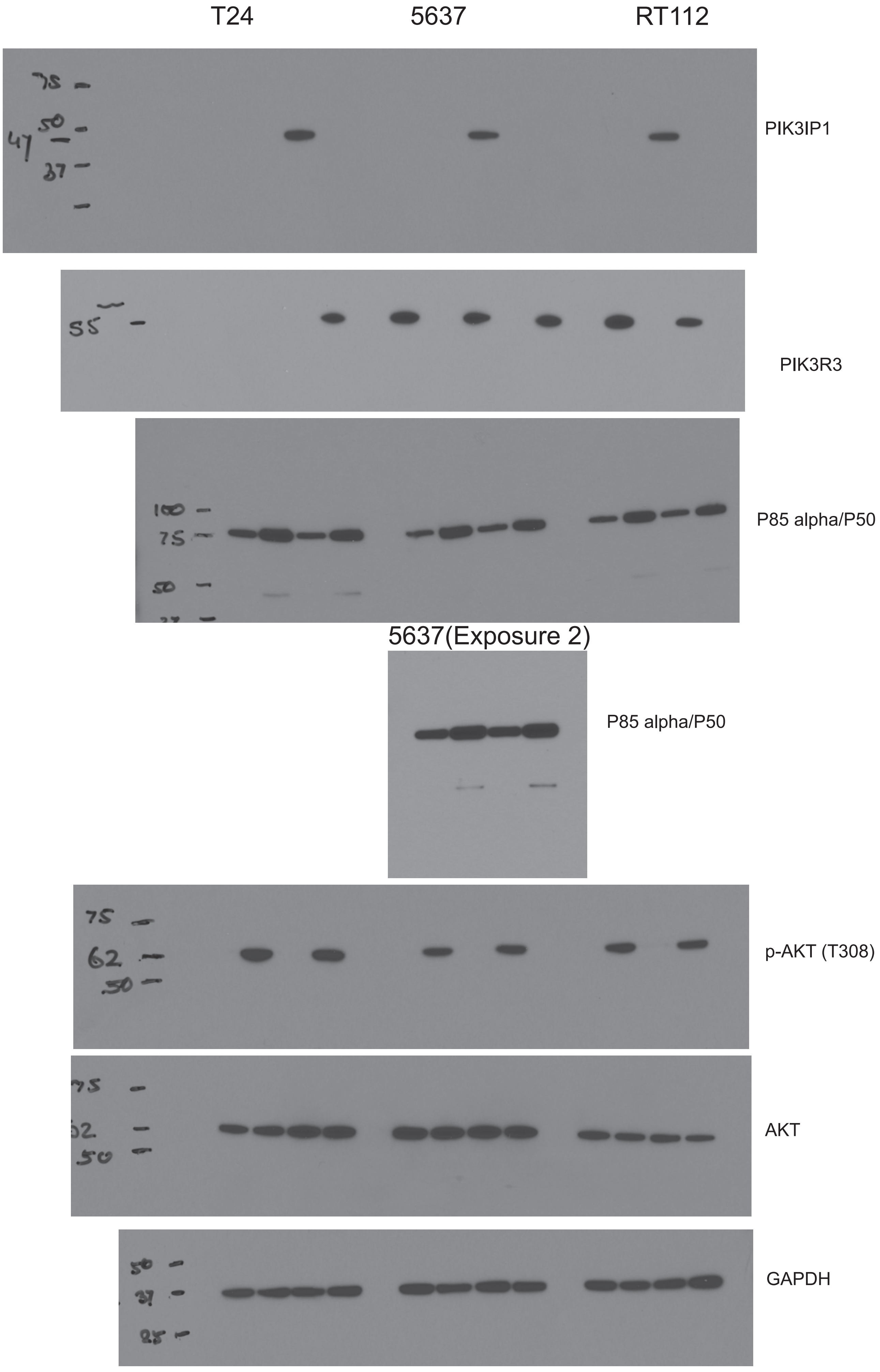

Uncut blot Figure 5C

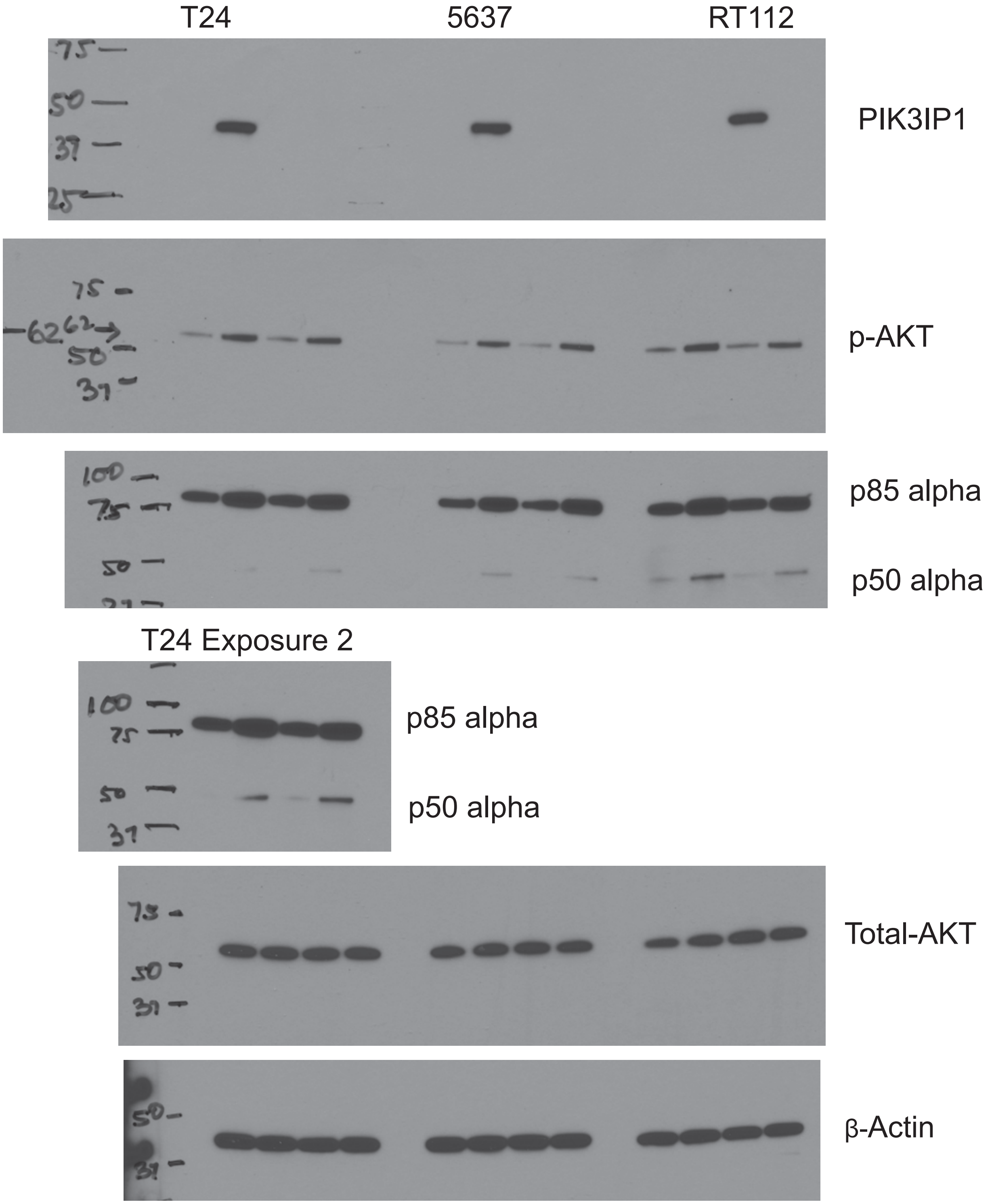

Uncut blot Figure 5E

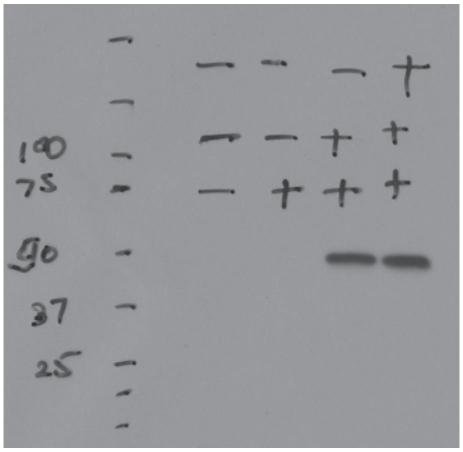

PIK3IP1

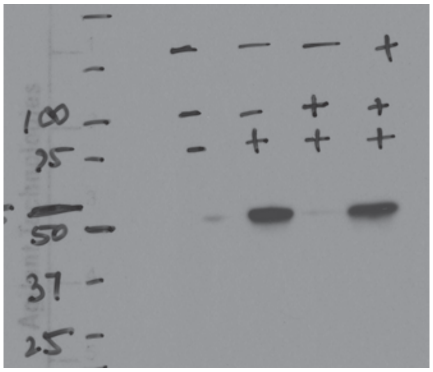

PIK3R3

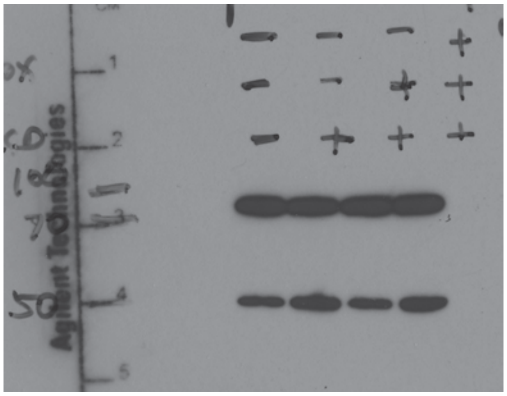

p85 alpha

p50 alpha

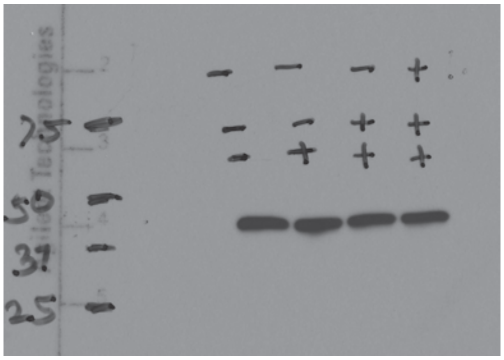

β-Actin

Uncut blot Fig 5F

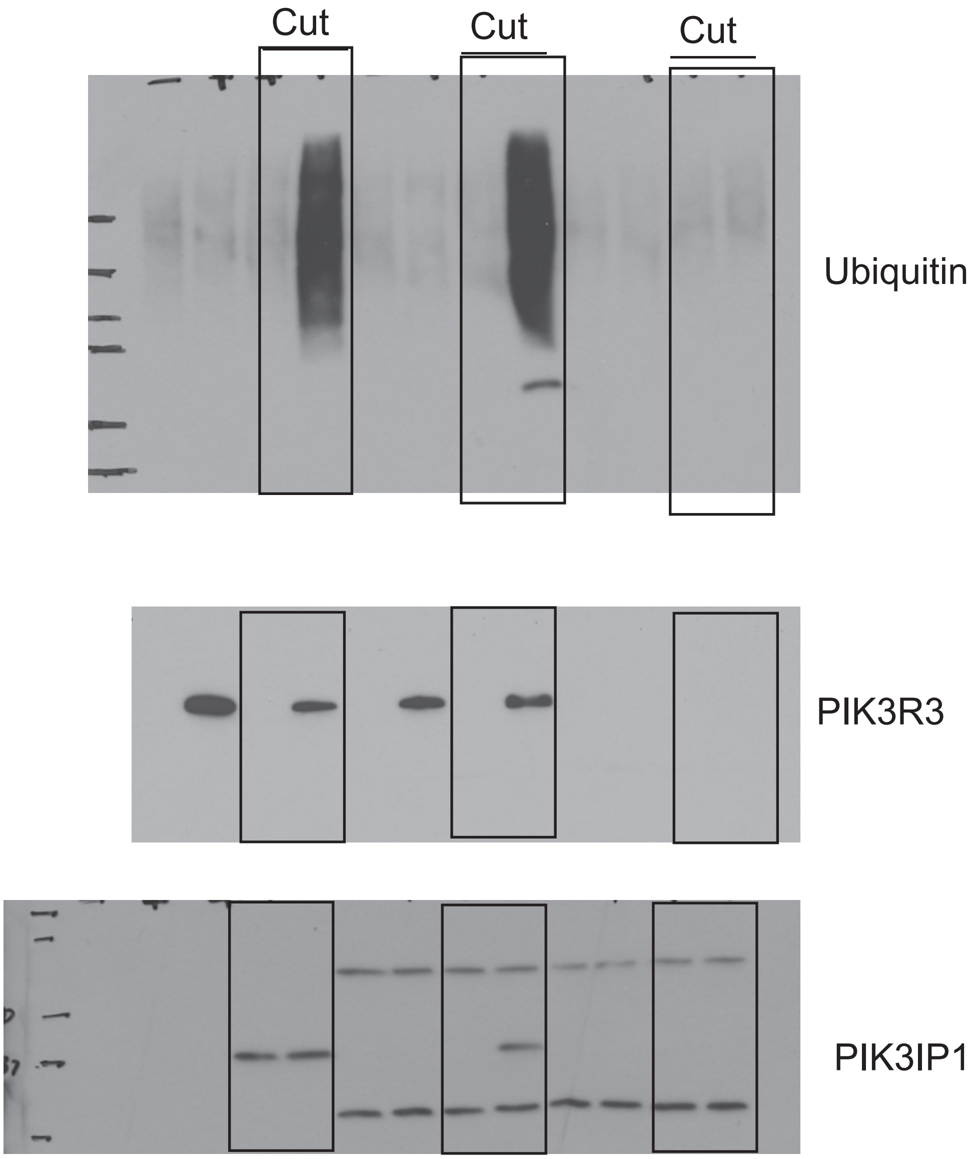

Uncut blot Figure 7A

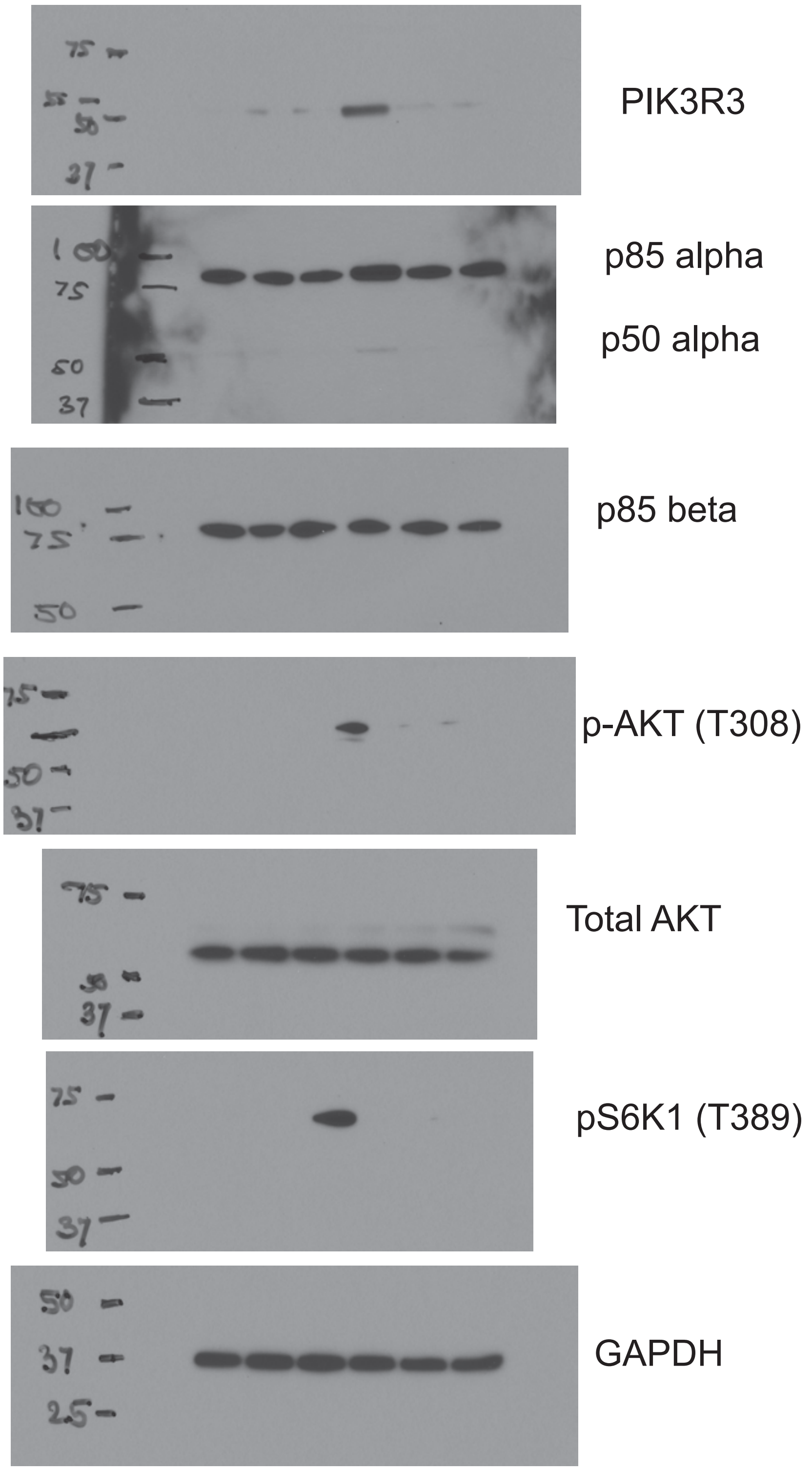

Uncut blot Figure 7C

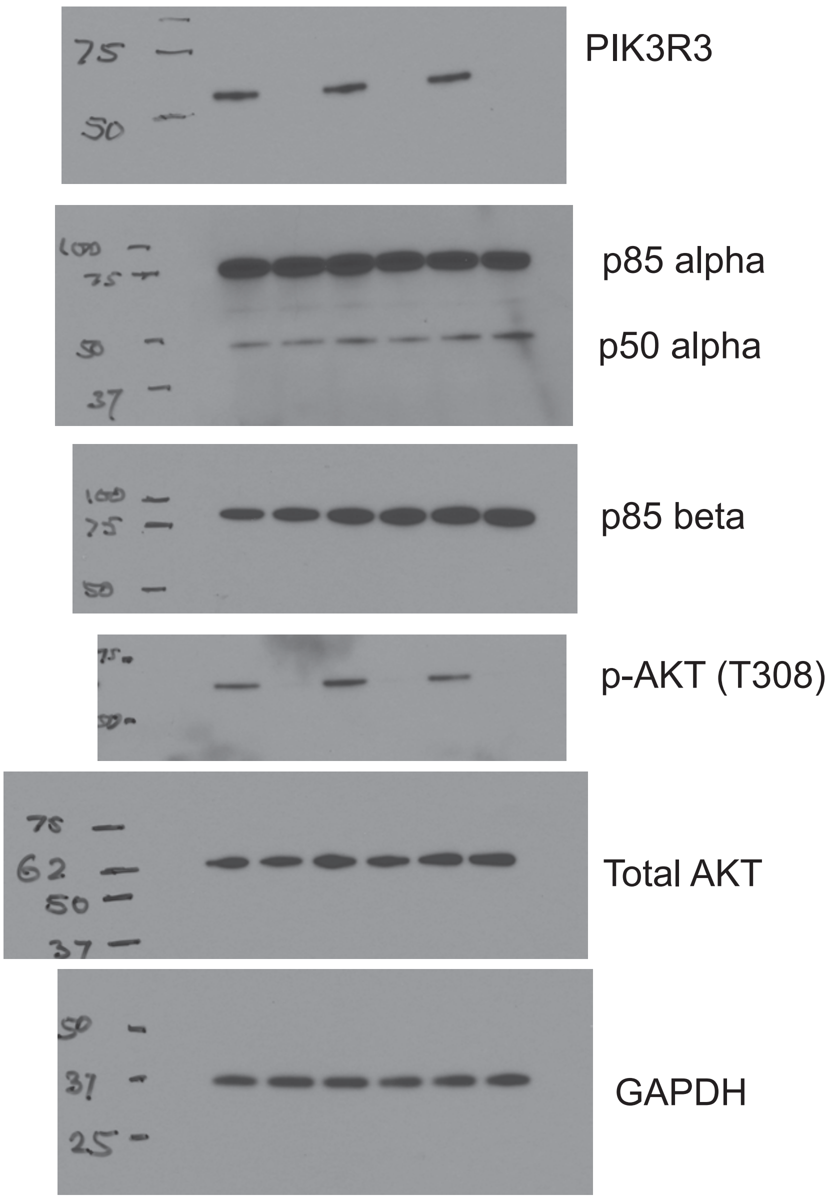

Uncut blot Figure 7E

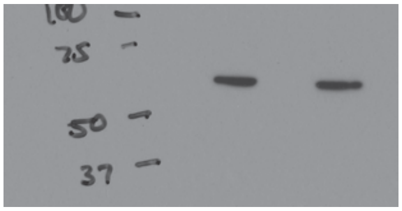

PIK3R3

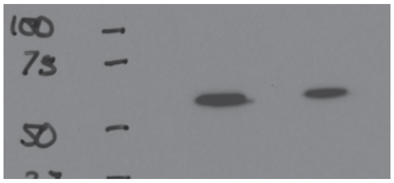

p-AKT (T308)

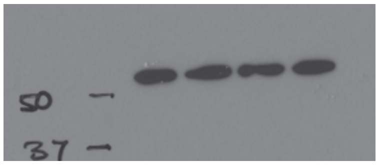

Total AKT

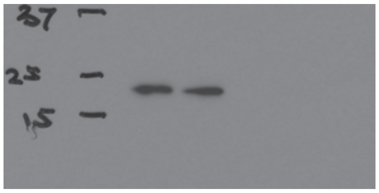

H3K27Me3

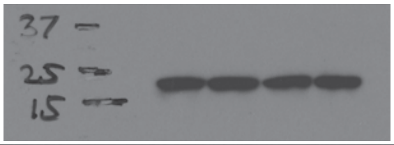

Histone H3

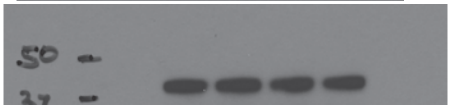

GAPDH

Uncut blot Figure S4D

CPI-1205

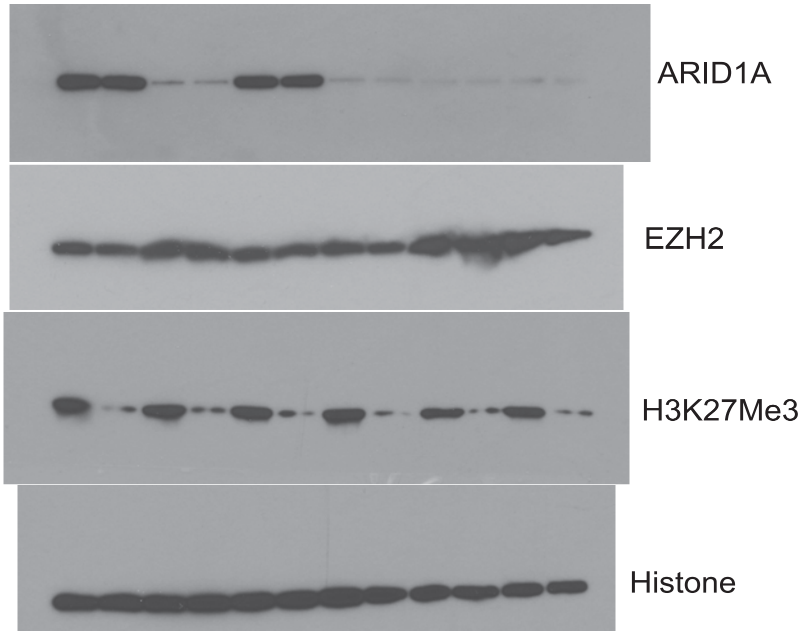

MAK-683

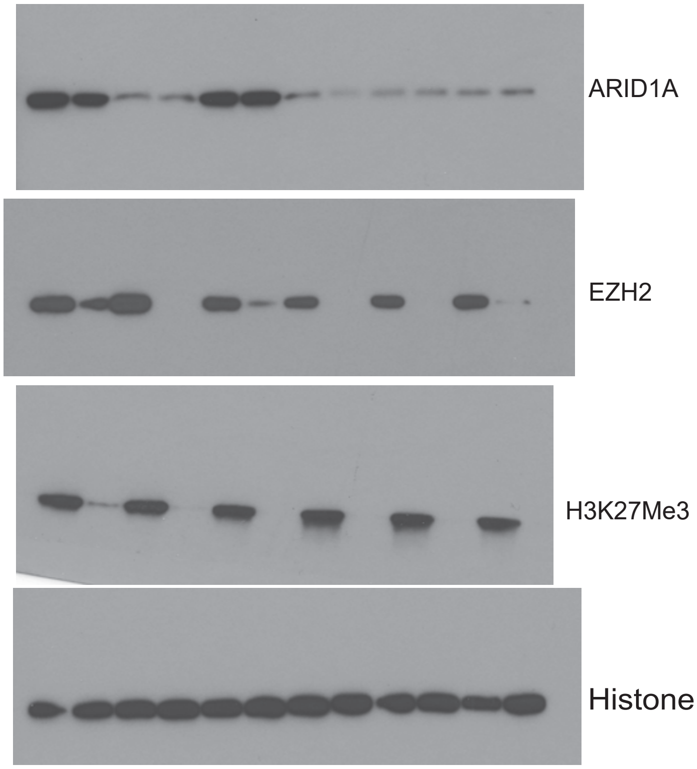

EPZ-6428

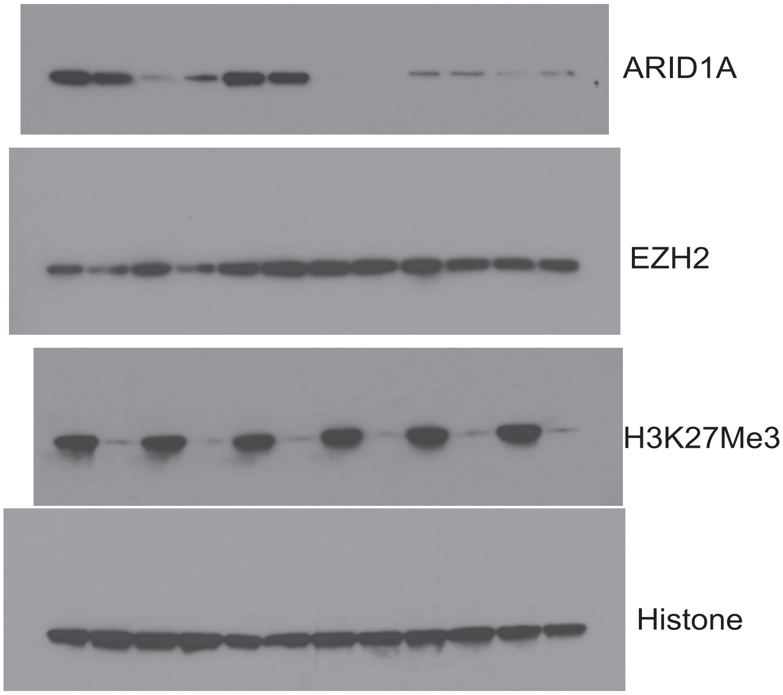

Supplement: Supplemental data [file jciinsight-7-155899-s252.pdf]
